# Nematocidal Effects of a Coriander Essential Oil and Five Pure Principles on the Infective Larvae of Major Ovine Gastrointestinal Nematodes In Vitro

**DOI:** 10.3390/pathogens9090740

**Published:** 2020-09-09

**Authors:** Mohamed A. Helal, Ahmed M. Abdel-Gawad, Omnia M. Kandil, Marwa M. E. Khalifa, Gareth W. V. Cave, Alison A. Morrison, David J. Bartley, Hany M. Elsheikha

**Affiliations:** 1 School of Veterinary Medicine and Science, Faculty of Medicine and Health Sciences, University of Nottingham, Leicestershire LE12 5RD, UK; ma.helal@nrc.sci.eg; 2 Department of Parasitology and Animal Diseases, Veterinary Research Division, National Research Centre, Giza 12622, Egypt; kandil_om@yahoo.com; 3 Parasitology Department, Faculty of Veterinary Medicine, Cairo University, Giza 12211, Egypt; a_abdelgwad@hotmail.com (A.M.A.-G.); marwwaehab40@yahoo.com (M.M.E.K.); 4 School of Science and Technology, Nottingham Trent University, Nottingham NG11 8NS, UK; gareth.cave@ntu.ac.uk; 5 Disease Control, Moredun Research Institute, Pentlands Science Park, Bush Loan, Penicuik EH26 0PZ, Edinburgh, UK; alison.morrison@moredun.ac.uk (A.A.M.); Dave.Bartley@moredun.ac.uk (D.J.B.)

**Keywords:** coriander essential oil, essential-oil-derived compounds, parasite control, gastrointestinal nematodes, larval motility assay, fluorometric assay

## Abstract

The anthelmintic effects of extracted coriander oil and five pure essential oil constituents (geraniol, geranyl acetate, eugenol, methyl iso-eugenol, and linalool) were tested, using larval motility assay, on the third-stage larvae (L3s) of *Haemonchus contortus, Trichostrongylus axei, Teladorsagia circumcincta, Trichostrongylus colubriformis, Trichostrongylus vitrinus* and *Cooperia oncophora*. Coriander oil and linalool, a major component of tested coriander oil, showed a strong inhibitory efficacy against all species, except *C. oncophora* with a half maximal inhibitory concentration (IC_50_) that ranged from 0.56 to 1.41% for the coriander oil and 0.51 to 1.76% for linalool. The coriander oil and linalool combinations conferred a synergistic anthelmintic effect (combination index [CI] <1) on larval motility comparable to positive control (20 mg/mL levamisole) within 24 h (*p* < 0.05), reduced IC_50_ values to 0.11–0.49% and induced a considerable structural damage to L3s. Results of the combined treatment were validated by quantitative fluorometric microplate-based assays using Sytox green, propidium iodide and C_12_-resazurin, which successfully discriminated live/dead larvae. Only Sytox green staining achieved IC_50_ values comparable to that of the larval motility assay. The cytotoxicity of the combined coriander oil and linalool on Madin–Darby Canine Kidney cells was evaluated using sulforhodamine-B (SRB) assay and showed no significant cytotoxic effect at concentrations < 1%. These results indicate that testing essential oils and their main components may help to find new potential anthelmintic compounds, while at the same time reducing the reliance on synthetic anthelmintics.

## 1. Introduction

Parasitic gastroenteritis has been increasingly recognized as a limiting factor for sustainable small ruminant farming globally due to the adverse impact on the health and productivity of the affected animals [1]. The efficacy of broad-spectrum anthelmintics, the mainstay tool used in helminth treatment and control plans, has been compromised by the multi-drug-resistant parasite populations [2]. This problem has been exacerbated by intensive management systems and improper parasite management practices such as inadequate or overuse of anthelmintics [3,4]. In addition, vaccination has not provided the expected level of protection in clinical trials against a number of nematode species [5]. Therefore, interest in searching for alternative and more sustainable approaches for controlling gastro-intestinal nematode (GIN) infections has been intensified [6]. Alternative approaches may offer the opportunity to reduce chemical residues, control anthelmintic resistant populations and make GIN control more affordable in limited-resource countries [7].

The anthelmintic activities of bioactive phyto-compounds, such as essential oils and their main components, have previously been examined [8]. These compounds possess biologically diverse functions, such as antioxidant, antimicrobial, analgesic, anti-inflammatory and anthelmintic activities [9]. Different essential oil extracts and some phenolic compounds from plants have been evaluated as potential anthelmintic agents for the treatment of various parasites [10]. For example, geraniol and geranyl acetate [11,12,13,14], eugenol and methyl iso-eugenol [15,16,17], and coriander [18,19], and linalool [9] have shown promising potential against *Haemonchus contortus* or *Caenorhabditis elegans*. Some components of essential oils have synergic properties when formulated in combinations [17,20]. More investigations are required to examine the anthelmintic efficacy of essential oils and their main constituents against a wide range of ruminant GINs.

Traditional methods used to assess the anthelmintic efficacy of chemical or natural substances involve using in vitro methods such as egg hatching assay [2], larval development assay [21], larval feeding inhibition assay, and larval exsheathment assay. Also, larval motility inhibition assay has been widely used in anthelmintic drug testing especially, in parasitic nematodes [22] and *C. elegans* [23]. Additionally, a quantitative colorimetric MTT assay has been developed for assessment of larval viability through measurement of metabolic activity of viable larvae [24]. This approach was optimized using larvae of *C. elegans* and first stage larvae (L1s) of *H. contortus* but has not been validated for third stage larvae (L3s). The feasibility of using fluorometric markers to evaluate larval viability or test drug efficacy has been reported [23]. Three fluorescent dyes (Sytox green, propidium iodide and C_12_-resazurin) have been extensively used in the evaluation of mammalian cell viability [25]. Sytox green and propidium iodide have been also utilized for in vitro drug screening using larvae of *C. elegans* [26,27] and resazurin was used for testing of *Trichuris muris* [28]. The application of high-throughput fluorometric methods on parasitic nematodes can provide a useful tool for the evaluation of nematode viability and identification of new compounds with anthelmintic properties.

In the present study, we investigated the potential larvicidal activities of essential oils and their main components against six GINs of small ruminants using a larval motility assay. We also developed three fluorometric microplate-based assays based on three fluorescent stains (Sytox green, propidium iodide and resazurin). These fluorometric assays were employed to validate the efficacy of the tested essential oils and their main components compared to the results obtained from larval motility assay. Scanning electron microscopy was used to characterize the ultrastructural cuticular damage that occurred in the L3s in response to treatment with the combination of most effective compounds identified in this study.

## 2. Results

### 2.1. Efficacy of Essential Oil Extracts Using Larval Motility Assay

**Figure 1:**
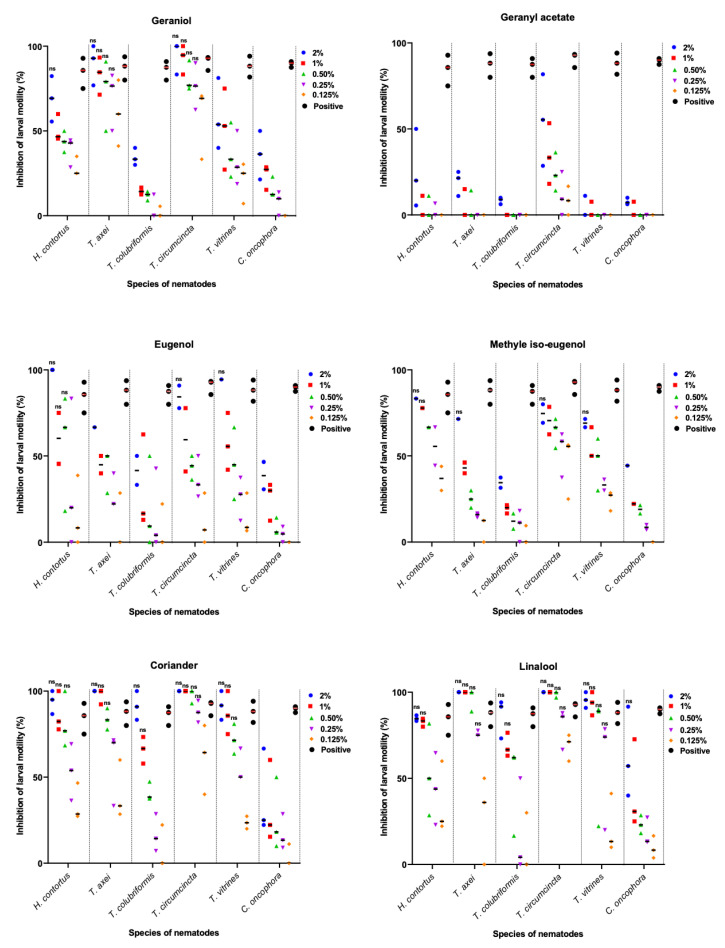
Inhibitory activity of a coriander essential oil and five pure principles (geraniol, geranyl acetate, eugenol, methyl iso-eugenol, and linalool) at the five indicated concentrations against the motility of L3s of six nematode species. Data represent the mean percentage of larval motility inhibition ± standard error of the mean (SEM) and were analyzed by one-way ANOVA (*p* < 0.05). ^ns^ No significant difference between treatment and positive control (20 mg/mL levamisole).

The results of individually evaluated compounds showed a significant (*p* < 0.05) inhibitory activity of coriander oil and linalool, the major component of coriander oil [29], against the motility of L3s of GINs at the highest concentrations tested for all parasite species, except for *C. oncophora* (Figure 1 and Video S1). Both coriander and linalool induced an inhibition of the motility of > 93% and 87% of the L3s at concentrations of 2% and 1%, respectively. Eugenol and methyl iso-eugenol exhibited only inhibitory effect at higher concentrations against *H. contortus, T. axei*, *T. vitrinus* and *T. circumcincta* L3s. Geraniol, was the least effective, as it reduced the motility of L3s of *H. contortus, T. axei* and *T. circumcincta* at 2% concentration by 82%, 90% and 94%, respectively. Geranyl acetate did not show any inhibitory activity. When considering the half maximal inhibitory concentration (IC_50_) of the essential oils, the IC_50_ values were relatively low for coriander oil (0.56 to 1.41%) and pure linalool (0.51 to 1.76%). Both exerted an inhibitory activity level comparable to that obtained by the positive controls (levamisole, 20 mg/mL).

**Figure 2:**
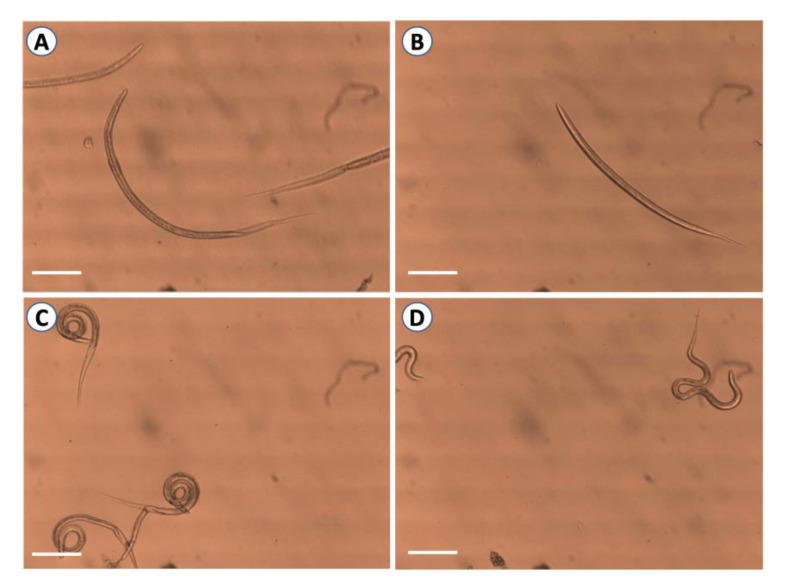
Microscopical examination of third larval stages of *H. contortus* after exposure to different treatments. (**A**) L3s treated with 2% coriander oil-linalool combination at 27 °C for 24 h. (**B**) Heat-treated L3s at 70 °C for 10 min as positive control 1. (**C**) L3s treated with 20 mg/mL levamisole as a positive control 2. (**D**) Untreated L3s used as a negative control. Dead L3s were immobile upon prodding in treated and positive control larvae. Negative control larvae were actively motile even after several days of incubation. Scale bar = 100 µm.

Next, we examined the anthelmintic activity of a combination of coriander oil and pure linalool at the five different concentrations against the larval motility of L3s of GINs (Table 1). The inhibition of larval motility was observed at lower concentrations, with at least 95% inhibition detected at 0.25% concentration for *T. circumcincta*, > 92% inhibition achieved with 0.5% concentration for *T. axei* and *T. vitrines*, and up to 80–87% reduction in larval motility of *H. contortus, T. colubriformis* and mixed larval suspensions at 1% concentration. These results were comparable to the efficacy of levamisole against different larval species (*p* > 0.05). The IC_50_ values of the combined treatment ranged from 0.11% to 0.49%, which were less than IC_50_ of individual essential oils. The combination index (CI) of the combined treatment was 0.54 to 0.79 for L3s of the six nematode species, suggesting the presence of a synergistic effect [30]. These data show that the coriander oil–linalool combination had a strong synergistic effect after 24 h of incubation with L3s and caused larval paralysis of all nematode species except *C. oncophora* larvae, when compared to the negative control. Microscopic examination of L3s was performed to monitor the inhibition in the motility of L3s. All treated L3s either by essential oils or by levamisole were immobile upon prodding. However, untreated L3s were actively motile even after several days of incubation (Figure 2 and Figure S1).

### 2.2. Optical Evaluation of the Feasibility of Fluorescent Stains

**Figure 3:**
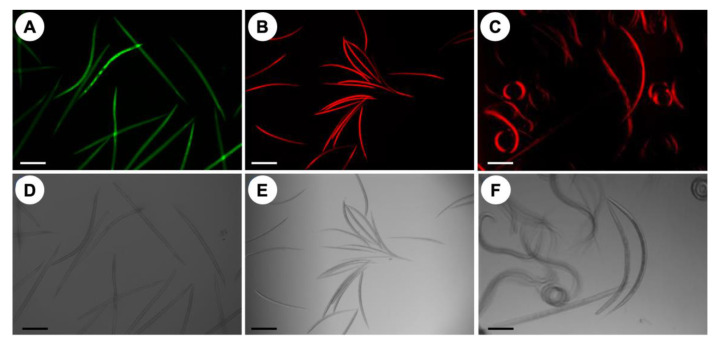
Representative images of the fluorometric assays. (**A**–**C**) The top row shows the fluorescent images, demonstrating the uptake of different fluorescent stains by L3s of *H. contortus*. (**A**) Sytox green and (**B**) propidium iodide staining of dead L3s, and (**C**) uptake of C_12_-resazurin by live L3s. (**D**–**F**) The bottom row shows the corresponding bright field images. Scale bar = 100 µm.

Microscopic analysis showed that Sytox green, propidium iodide and C_12_-resazurin stains enabled the differentiation between live and dead L3s. However, Sytox green displayed higher fluorescence intensity for dead L3s than the PI stain (Figure 3). C_12_-resazurin was able to show viable L3s, which emitted red fluorescence, whereas L3s killed by treatment with 50% methanol, did not show any red fluorescence (Figure 3).

### 2.3. Optimization of the Fluorometric Assays

**Figure 4:**
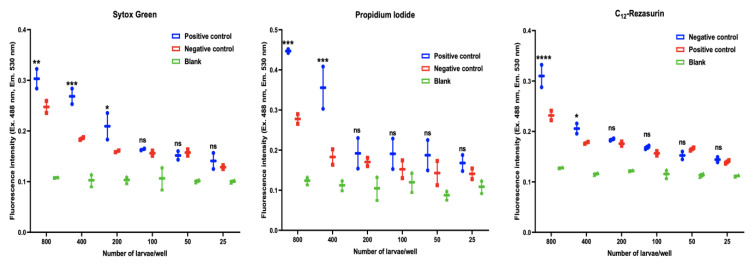
Fluorescence intensity of serially diluted dead-live L3s based on Sytox green, propidium iodide and C_12_-resazurin staining. Positive control (treated with 50% methanol), negative control (live L3s) and blank (L3s-free). Results are expressed as the mean of intensity ± SEM (* *p* < 0.05; ** *p* < 0.01; *** *p* < 0.001; **** *p* < 0.0001 compared to control groups; ns = non-significant).

We determined the fluorescence intensities produced by serially diluted live/dead L3s compared to blank wells (without larvae). After 24 h of incubation, the fluorescence signals correlated with the number of larvae/well over the entire range examined, from 25 to 800 larvae/well (Figure 4). The fluorescence signals from the positive control wells containing dead L3 were greater than signals produced by live larvae present in the negative control wells in case of staining with Sytox green and PI with correlation coefficient (between larval count and florescence signals) of 0.92 and 0.84, respectively (*p* = 0.05). In regard to C_12_-resazurin, viable L3s emitted more fluorescence signals than that produced by dead L3s treated by 50% methanol with 0.91 correlation (*p* = 0.05). The fluorescence intensities became less discriminatory when a low number of L3s was used, where at 25 larvae/well similar values were obtained in positive control, negative control and blank wells. The sensitivity of the fluorometric assays was improved when a larger number of L3s (800 and 400 larvae/well) (*p* < 0.05) were used, except the Sytox green, which showed significant correlations even with using down to 200 larvae/well (Figure 4).

### 2.4. Efficacy of Coriander Essential Oil -Linalool Combination Based on Fluorometric Assays

Table 2 and Table 3 show the fluorescence uptake by L3s treated with five concentrations of a combined coriander-linalool preparation compared to positive control (50% methanol-treated L3s), negative control (live L3s) and blank (without L3s) based on Sytox green and PI staining, respectively. Overall, wells treated with higher concentrations of coriander essential oil–linalool combination showed higher fluorescence signals and the strength of the fluorescence decreased in wells having lower oil concentrations. In regard to C_12_-resazurin staining, fluorescence intensities of wells containing L3s exposed to higher concentrations of coriander essential oil–linalool combination were lower than intensities measured from wells with low concentrations (Table 4). Clear statistical differences between the three fluorometric assays indicate that these assays have adequate sensitivity to differentiate between live and dead L3s, and between treated, untreated (control) and blank wells. With the exception of *C. oncophora*, dead L3s of other GINs incubated with combined coriander oil- linalool at concentrations 2% and 1% produced more fluorescence compared to untreated L3s and were not-significantly different from positive control samples *(p* < 0.05), when Sytox green and PI were used (Table 2 and Table 3). On the other hand, with C_12_-resazurin stain only 0.125% concentration resulted in significantly more fluorescence intensity from live L3s than other concentrations when compared to negative control (*p* < 0.05) (Table 4). The nonlinear correlations (R^2^) for these fluorometric data suggest a dose-dependent relationship. Sytox green was the most sensitive in detecting the inhibitory effect of the combined coriander oil-linalool and provided the highest R^2^ values for all tested nematode species except for *T. circumcincta* and *T. axei* (Table 2). In addition, as shown in Table 2, Table 3 and Table 4, the Sytox green showed the lowest IC_50_ values followed by PI, whereas C_12_-resazurin yielded more variable values.

### 2.5. Efficiency of Fluorescence Stains Versus Larval Motility Assay

**Figure 5:**
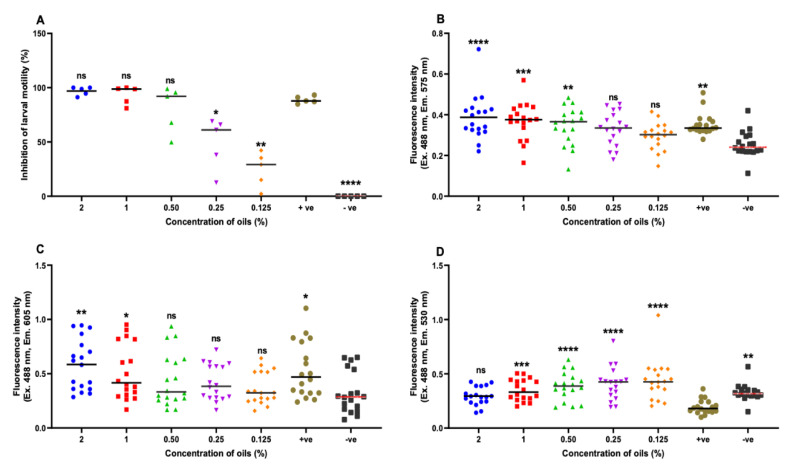
Comparative performance of fluorescent stains versus larval motility assay. (**A**) Percentage of inhibition of motility of L3s of mixed GINs. L3s were treated with coriander oil-linalool combination at the five indicated concentrations. Positive control (L3s treated with 20 mg/mL levamisole) and negative control (L3s in PBS-Tween 2%). (**B**–**D**) Fluorescence intensity of L3s of mixed GINs stained with (**B**) Sytox green, (**C**) PI, and (**D**) C_12_-resazurin, respectively. Positive control (L3s treated with 50% methanol), negative control (live L3s) and blank (without L3s). Results are expressed as the mean ± SEM (* *p* < 0.05; ** *p* < 0.01; *** *p* <0.001; **** *p* < 0.0001 compared to control groups). ns = non-significant); +ve = positive control; –ve = negative control.

We detected a correlation in the IC_50_ values between the results obtained by larval motility assay (Table 1) and Sytox green staining assay (Table 2) when investigating the efficacy of coriander oil-linalool combinations against individual GIN species. A similar trend was observed when a larval mixture of different nematode species was examined using the larval motility assay (Figure 5A) and fluorometric assay using Sytox green (Figure 5B). However, the results of fluorometric assays employing PI (Table 3) or C_12_-resazurin (Table 4) did not correlate with those obtained by larval motility assay (Table 1) when single larval suspensions were treated with coriander oil-linalool combination. The results based on larval motility (Figure 5A) significantly outperformed results obtained by the PI and C_12_-resazurin stains (Figure 5C,D), as shown by the significant difference in the IC_50_ values.

### 2.6. Scanning Electron Microscopy (SEM)

**Figure 6:**
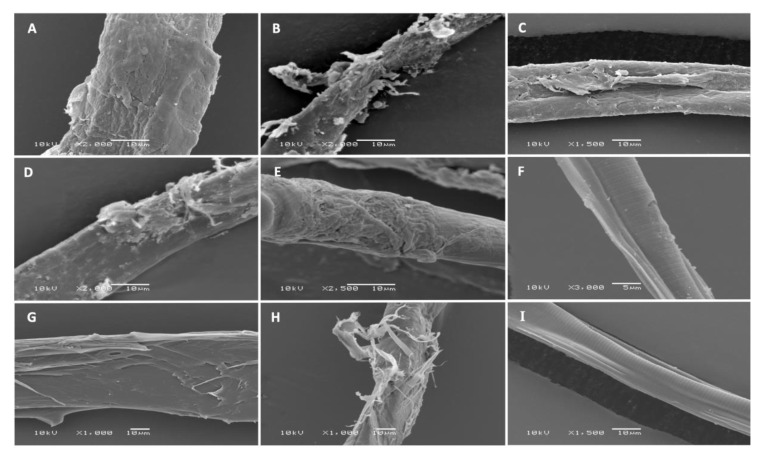
SEM micrographs of L3s treated with 2% coriander oil + linalool combination for 24 h at 27 °C. (**A**) *Haemonchus contortus*, (**B**) *Trichostrongylus axei*, (**C**) *Trichostrongylus colubriformis*, (**D**) *Teladorsagia circumcincta*, (**E**) *Trichostrongylus vitrinus* and (**F**) *Cooperia oncophora*. Positive controls included L3s of *H. contortus* killed by exposure to 70 °C in water bath for 10 min (**G**) or treated with 50% methanol for 24 h (**H**). (**I**) Negative control includes L3s exposed to the solvent PBS-Tween 2%.

SEM micrographs of L3s of all six nematode species exposed to a combined coriander oil and linalool treatment are shown in Figure 6. L3s incubated with the control solvent showed no morphological or structural alterations. However, L3s treated with coriander oil and linalool combination showed significant damage and loss in the cuticle in all examined GIN, except L3s of *C. oncophora*. These morphological alterations appeared similar to the damage observed in 50% methanol-treated L3s, but not the heat-killed L3s, which had more severe damage that involved the deeper muscular layer of L3s.

### 2.7. Cytotoxic Effects of Coriander and Linalool

**Figure 7:**
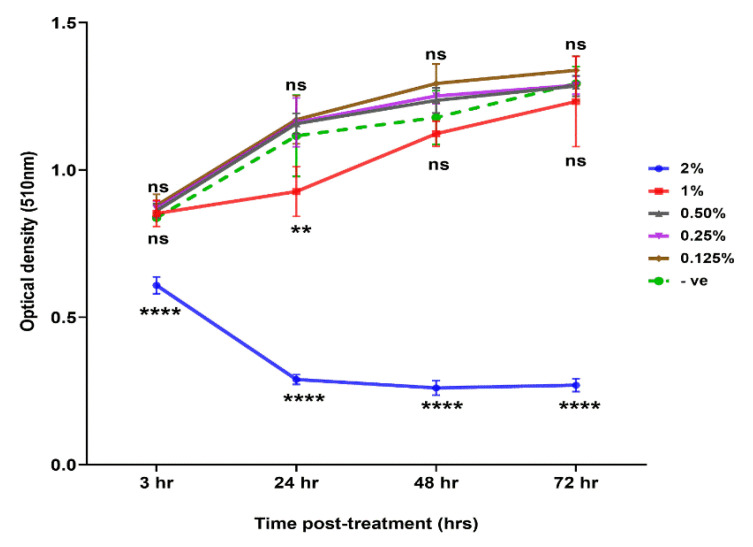
Cytotoxic effect of combined coriander oil and linalool on MDCK cells measured by the sulforhodamine B colorimetric assay. Results are expressed as the means of optical density (510 nm) ± standard errors, showing significant difference by one-way ANOVA followed by Tukey’s post-hoc test (** *p* < 0.01; **** *p* < 0.0001 compared to negative control; ns = non-significant).

The coriander oil and linalool combination at 2% concentration showed a cytotoxic effect on MDCK cells in a dose- and time-dependent manner (Figure 7). However, the cytotoxic effect of this combination was not significantly different from the control at concentrations < 1%. The effective concentration (EC_50_) was calculated as 1.17%, which was higher than IC_50_ values of coriander oil and linalool combinations (0.11–0.49%).

## 3. Discussion

Various studies have demonstrated the anthelmintic activity of some essential oils and their main components against GINs especially *H. contortus* [10,21,31,32,33,34]. In the present study, we evaluated the activity of a coriander essential oil whose main components were linalool (68.03%), α-tinene (9.71%), γ-terpinene (10.48%) and camphor (11.76%) (Figure S2), and five common essential oil components, namely geraniol, geranyl acetate, eugenol, methyl iso-eugenol, and linalool, against the motility of L3s of *H. contortus, T. axei, T. colubriformis, T. vitrinus, T. circumcincta* and *C. oncophora*. Our results showed that coriander oil and linalool had the most potent inhibitory effect against larval motility of five GIN nematode species. Eugenol and methyl iso-eugenol inhibited larval motility of four species. The geraniol had larvicidal efficacy against only three species, but geranyl acetate did not show any inhibitory effect. The efficacy of extracts of some plants has been attributed to the abundance of some of these compounds. For example, linalool was a constituent of a hydro-alcoholic extract of *Coriandrum sativum* seeds showing ovicidal properties against *H. contortus* eggs in vitro [18]. The bioactive compound of *O. gratissimum*, eugenol, inhibited the hatching of *H. contortus* eggs [16]. Other studies identified anthelmintic potential of geraniol against sheep GINs in vitro [12,13,14].

Limited knowledge is available concerning the anthelmintic activity of essential oil and their main components against GINs. The present study offers a more comprehensive overview about the efficacies of some essential oil components and compares them to a coriander essential oil against six different GIN nematode species. The mode of action of coriander oil and linalool is related to disruption of membrane function [35,36,37] through strong lipolytic activity [38]. Linalool was thought to cause neurotoxicity through inhibition of the nematode acetylcholine receptors [39]. The lack of effect of these compounds on *C. oncophora* larvae may depend on the impermeability of their cuticle to these compounds [18]. This observation may not be surprising given that *Cooperia* species has been identified as one of the dose-limiting species for some anthelmintics, particularly the macrocyclic lactones [40,41], and given the high prevalence of resistance in *C. oncophora* to anthelmintic drugs compared to other GIN species [42,43]

One interesting finding was the synergistic inhibitory effect of both coriander oil and linalool on larval motility, which was consistent across trichostrongylid species even when tested against mixture of these species. These results support the previous hypothesis regarding the possible synergistic effect of combining essential oil main constituents [6]. Several reports have shown additive nematocidal interactions of binary, tertiary or quaternary compounds from the same or different chemical groups of essential oil main constituents [44]. This was exemplified in the demonstration of potent inhibition of egg hatching of a multi-drug resistant strain of *H. contortus* through combination of cinnamaldehyde + carvacrol and anethole + carvone [17]. Coriander oil has been reported to have nematocidal properties against the pine wood nematode; *Bursaphelenchus xylophilus* [38] and have insecticidal activity against adults of *T. castaneum*, *S. oryzae*, and *L. serricorne* [45]. Additionally, coriander oil was effective against bacterial isolates in combination with antibiotics [46]. These findings suggest that a complex interaction between different essential oil main constituents may have produced a synergistic effect and may be attributed to monoterpenoids of coriander oil, comprising of linalool (68.03%), α-tinene (9.71%), γ-terpinene (10.48%) and camphor (11.76%) (Figure S2). Previous studies have shown anthelmintic activity of essential oil derivatives from *C. sativum* on the motility of *H. contortus* adults [18] or against larval development with 97.8% inhibition at 10 mg/mL [19].

SEM analysis revealed considerable damage to the cuticle and loss of the structural integrity of all treated larvae, but not of *C. oncophora* (Figure 5B). The cuticles of different nematode species differ in complexity and composition; which may influence their physiological responses [47]. These results agree with the previous findings regarding the anthelmintic effect of grape pomace extract against GINs [48]. Coriander oil and linalool, are reported to affect membrane diffusion through alteration of electrostatic property and permeability of cuticle in addition to inhibition of cuticular protein [49,50]. Therefore, the cytotoxicity of combined coriander oil and linalool on MDCK cells observed at > 1% concentration may be attributed to disruption of cell membrane integrity. We did not determine the in vivo toxicity of the tested oils or investigate their effect on ruminal microbiota. However, oral administration of essential oil components, such as linalool, geraniol and geranyl acetate did not alter the hematological parameters, body weight or tissue of treated compared to control rodents [14,31]. Some essential oils and their components, especially terpenes have been shown to have antibacterial activity and can alter bacterial growth including ruminal microflora [51]. However, a mixture of thymol, eugenol, vanillin, guaiacol, and limonene had a modest effect on ruminal fermentation and ruminal microbiome, probably due to adaptation of ruminal microbiota to essential oil components [52]. A reduction in methane production was detected when animals were supplemented with a blend of three monoterpoids (eugenol, geranyl acetate and coriander oil) [53].

The inhibition of larval motility, as an indicator of worm viability after exposure to molecules or substances, is generally used in drug screening [23] and as a gold standard for testing anthelmintics [54]. However, this assay seems to be impractical especially when evaluating numerous compounds or concentrations [24]. More throughput assays were developed and used in non-parasitic nematodes (*C. elegans*) to provide a more convenient means to determine the worm viability after treatment with drugs [55]. In this study, we used three fluorescent strains (Sytox green, PI and C_12_-resazurin) to identify dead/live larvae and tested their feasibility for testing compounds examined in this study. Initially, we optimized the number of larvae that was sensitive enough to discriminate between positive control (50% methanol-treated L3s), negative control (live non-treated L3s) and blank wells for the three stains. The fluorescence intensities correlated with the number of larvae down to 400 larvae/wells for PI and C_12_-resazurin, but down to 200 larvae/well for Sytox green. These results seem to agree with those reported previously, where fluorescence signals were proportional to the number of larvae and significance differences were detected when at least 500 larvae were tested [26]. The results obtained in the present study showed the usefulness of these fluorescent stains as a rapid screening approach for testing the viability of L3s after exposure to essential oil and their components. However, compared to other two stains, Sytox green produced the most reliable IC_50_ result and in a dose-dependent manner similar to that obtained by larval motility inhibition assay (Table 1, Figure 5A), against individual species (Table 2) and mixed species (Figure 5B). The use of Sytox green for rapid assessment of larval viability has been previously suggested [56] where heat-killed nematodes were found to emit a strong fluorescence intensity after uptake of Sytox green.

## 4. Materials and Methods

### 4.1. Coriander Essential Oil and Pure Principles

Coriander oil and five pure principles, including geraniol, geranyl acetate, eugenol, methyl iso-eugenol, and linalool, were obtained from Bristol Botanicals Ltd. (Bristol, UK). To improve emulsification and prepare the required concentrations, the oils were diluted in phosphate buffered saline (PBS) + 2% Tween 20 (*v*/*v*) and the mixture was vortexed until the oil-solvent-PBS mixture formed a stable emulsion [16]. The oils were tested individually at serial concentrations (2%, 1%, 0.5%, 0.25% and 0.125% *v*/*v*). Coriander oil and linalool that showed the highest efficacy against different nematode species were mixed in a 1:1 ratio and their synergistic effect was evaluated at the same above concentrations.

### 4.2. Source of Nematode Larvae

L3s of six GIN species (*H. contortus, T. axei, T. colubriformis, T. vitrinus, T. circumcincta* and *C. oncophora*) used in this study were kindly provided by Alison Morrison (Moredun Research Institute, Scotland). The larvae were cultured [57] from feces collected from donor sheep mono-specifically infected by each species for 7–12 days, depending on the nematode species. L3s were stored in tap water between 4–8 °C in a vented untreated 75cm^2^ culture flask (Sarstedt Ltd., Nümbrecht, Germany). The larvae were washed by centrifugation at 700× *g* for 5 min, followed by two washes with PBS and only active motile larvae were used in the study. A mixed larval population was prepared by mixing equal numbers of L3s of all six GIN species.

### 4.3. Larval Motility Assay

The evaluation of the larval motility was based on the procedures described previously [32]. In a 48-well tissue culture plates, an equal number of L3 suspension (100 larvae/200 μL) of individual or mixed GIN species were exposed to individual oil compounds at different concentrations (2, 1, 0.5, 0.25, or 0.125% *v*/*v*). Each plate included two positive controls (L3s treated with levamisole 20 mg/mL in PBS-2% Tween 20 and ‘dead’ larvae, heat-killed by exposure to 70 °C in a water bath for 10 min) and two negative controls (L3s treated with PBS-2% Tween 20 or distilled water). The plates were incubated at 27 °C for 24 h and the numbers of mobile and immobile larvae were counted using a light microscope (Leica Microsystems, Milton Keynes, UK). The larvae were considered motile when they exhibit a mild sinusoidal mobility accelerated by plate agitation and exposure to a light source. The combination of the two most potent oil compounds were tested using the same protocol used for testing the individual oil compounds. All the assays were performed independently three times and in three technical replicates. Results were expressed as percentage inhibition of larval motility of tested L3s compared to control L3s.

### 4.4. Development of Fluorometric Microplate-based Assays

We developed three fluorescence microplate-based assays in order to evaluate the viability of L3s following exposure to essential oils using Sytox green, propidium iodide (PI) and C_12_-resazurin. These three fluorescent dyes were prepared according to the manufacturer’s instructions (Molecular Probes Inc., Invitrogen detection technologies, USA). The working concentrations for Sytox green (membrane-impermeable DNA dye), PI (membrane-impermeable DNA dye), and C_12_-resazurin (metabolic activity dye) were 1, 20 and 50 µM, respectively [27].

#### 4.4.1. Optimization of the Fluorometric Microplate-based Assays

We determined the optimal number of L3s that can provide a sufficient level of sensitivity to discriminate live and dead L3s. For each assay, a suspension of live or dead L3s of different nematode species were serially diluted (800, 400, 200, 100 and 50 L3s/100 µL PBS) and added into 96-well microtiter plate (triplicate wells/dilution). Dead L3s were obtained either by heat exposure of L3s at 70 °C in a water bath for 10 min or by treatment with 50% methanol in PBS. The 50% methanol was used as a positive control because it kills the L3s rather than paralyzing them. The remaining wells of the plates were left empty as blank controls containing 100 µL of PBS without L3s in order to evaluate the correlation between the number of L3s and the uptake of stain (i.e., fluorescence signals emitted from live and dead L3s) in comparison with background signals from control and blank wells. The plates were incubated at 20 °C for 24 h, followed by addition of 1 µL/stain/per well, and incubation of microplates at room temperature for 15 min on a horizontal shaker at 120 rpm. The fluorescence intensity was recorded at excitation 488 nm using microplate reader (Varioskan Flash, Thermo Fisher Scientific Inc., Waltham, MA, USA) with emission at 575 nm for Sytox green, 605 nm for PI and 530 nm for C_12_-rezasurin.

#### 4.4.2. Efficacy of a Coriander Essential Oil and Five Pure Principles Using Fluorometric Assays

The three fluorometric assays were performed with some modifications from the methods described previously for *C. elegans* [26]. Briefly, a final volume of 50 μL of L3s were added to a 96-well microtiter plate (600 L3s/well), followed by addition of equal volume of two-fold serial dilutions of combined coriander oil plus linalool (final concentrations; 2%, 1%, 0.5%, 0.25%, and 0.125%). The negative control was PBS-2% tween 20% and 50% methanol was used as positive control [58]. Three separate experiments, each with three technical replicates, were carried out for each of the above concentrations and for the controls. The plates were incubated in an incubator at 20 °C for 24 h, then centrifuged at 800× *g* for 10 min to pellet the L3s. The L3s were washed three times using distilled water to remove essential coriander oil and linalool before staining. A final volume of 1 µL of Sytox green, PI and C_12_-resazurin at the same concentrations mentioned above were added per well followed by incubation of microplates at room temperature for 15 min on a horizontal shaker at 120 rpm. Fluorescence intensities were measured using microplate reader (Varioskan Flash, Thermo Fisher, UK) as described above. Representative images were acquired with a Leica DME 5000b fluorescence microscope (Leica Microsystems, Milton Keynes, UK), using appropriate filters for fluorescein isothiocyanate (FITC) and tetramethyl rhodamine isothiocyanate (TRITC).

### 4.5. Scanning Electron Microscopy (SEM)

The L3s of *H. contortus, T. axei, T. colubriformis, T. vitrinus, T. circumcincta* and *C. oncophora* were treated with 2% of combined coriander oil–linalool at 27 °C. L3s of *H. contortus* exposed to heat treatment or 50% methanol were used as positive controls, or to the solvent (PBS-2%Tween 20) and served as negative controls. Twenty-four hours after treatment, L3s from each group were pooled into 1.5 mL Eppendorf tubes, rinsed 3 times with distilled water and centrifugation at 600× *g* for 5 min. Subsequently, L3s were fixed in 3% glutaraldehyde/0.1M cacodylate buffer (CACO; *w*/*v*) for 24 h. This was followed by washing twice with 0.1 M CACO buffer (pH 7.4) and fixation for 1 h in 1% osmium tetroxide diluted in 0.1 M CACO buffer. Samples were then washed three times with distilled water and dehydrated in a graded series of ethanol (70%, 80%, 90% and 100%); each 10 min twice. After dehydration, samples were immersed in hexamethyldisilazane (HMDS) (Acros, Germany) for 5 min twice to further enhance the drying of the samples whilst maintaining the structure of the L3s. This was followed by critical point drying in liquid carbon dioxide using an EMS 850 critical point drying apparatus. Finally, the L3s were mounted on aluminum SEM stubs (25 mm) supported with a double-sided carbon sticker, inserted into the coating crucible, flushed with argon and coated with 10 nm layer of gold and visualized with a scanning electron microscope (FEI Quanta 200 3D dual Beam FIB-SEM, Eindhoven, Netherlands) at an accelerating voltage of 10 kV.

### 4.6. Cytotoxicity Assay

Madin–Darby Canine Kidney cells (MDCK) were obtained from the European Collection of Authenticated Cell Cultures (ECACC, Salisbury, UK) and were maintained in Dulbecco’s Modified Eagle Medium (DMEM; Gibco^TM^) supplemented with 5% inactivated fetal bovine serum (FBS), 1% glutamine (*v*/*v*), penicillin (100 U/mL) and streptomycin (100 µg/mL) (Sigma-Aldrich). Cultures were incubated at 37 °C and 5% CO_2_ in a humidified atmosphere. Confluent cells were trypsinized and plated at a density of 6 × 10^4^ cells/100 µL/well into 96-well tissue culture microtiter plates for 24 h. Blank wells contained only DMEM. Combined coriander oil and linalool were solubilized in PBS-Tween 20, and serially diluted with culture medium to obtain concentrations that ranged from 2% to 0.125% (*v*/*v*). The medium was then discarded and replaced with tested medium containing various concentrations in triplicate (100 µL/well). The microplates were sealed and incubated for 3, 24, 48 and 72 h. Control wells contained the diluent only (PBS-Tween 20). Cytotoxicity evaluation was performed using the sulforhodamine-B (SRB) assay as described previously [59]. Briefly, at the end of each incubation time, cells were fixed with cold 10% trichloroacetic acid (TCA, 25 µL/well) and incubated at 4 °C for 1 h. The TCA was discarded, and the plates were washed gently three times with tap water and left to dry at room temperature. The sulforhodamine-B dye (SRB 0.05% wt./v in 1% acetic acid (Sigma-Aldrich, Irvine, UK) was added to each well (25 µL/well) and incubated for 15 min and plates were kept in a dark place. The plates were washed three times with 1% acetic acid solution to remove unbound dye and left to dry. Bound stain was solubilized by adding 150 µL/well of 10 mM Tris base solution (pH 10.5). The plates were agitated in a shaker for 5 min to solubilize the protein-bound dye thoroughly before reading. The optical density (OD) of the wells was measured at 510 nm using a microplate reader (Biotek Instruments Inc., Powerwave XS, Winooski, VT, USA). Absorbance values were corrected for background absorbance values from blank wells containing medium only and compared with absorbance values of negative control wells. All experiments were performed three separate times each in triplicate.

### 4.7. Statistical Analysis

All statistical analysis was performed using GraphPad Prism version 8.0 for Windows (GraphPad Software, USA). The results of larval motility assay, fluorescence assays and cytotoxicity assay were analyzed using one-way Analysis of Variance (ANOVA) and Tukey’s test. A *p* < 0.05 was deemed as a statistically significant. Sigmoidal inhibition dose–response curves were calculated using a variable slope nonlinear regression model. A four-parameter logistic equation was applied using global curve-fitting, with the bottom of the curves constrained to zero. For each treatment, the half maximal inhibitory concentration (IC_50_) and R^2^ values were calculated. The synergistic effect of coriander oil and linalool combination was analyzed using CompuSyn Version 1.0 [30] to calculate the combination index (CI), with CI <1, =1, and >1 indicates synergism, additive effect, and antagonism, respectively.

## 5. Conclusions

The coriander oil and five common essential oil constituents exhibited a significant anthelmintic activity against L3s of five major GIN species. The coriander oil and linalool showed the most inhibitory effects against L3s. Combined treatment using coriander oil enhanced with additional linalool offered more synergistic effect on larval motility indicated by the extensive cuticular damage of treated L3s. We also optimized three quantitative fluorometric assays for evaluation of the larval viability after treatment with tested compounds. The agreement between Sytox green and larval motility assay makes a fluorometric microplate assay based on Sytox green a valuable alternative approach for high-throughput anthelmintic drug testing. More comprehensive studies are required to assess the bioavailability and safety of the tested coriander oil and linalool in sheep. Investigation on the efficacy of treatments combining these compounds with anthelmintic drugs is also warranted.

## Figures and Tables

**Table 1:** Inhibitory efficacy of combined coriander oil-linalool treatment at five different concentrations on the motility of L3s of six nematode species.

F.C. (%)	*H. contortus*	*T. axei*	*T. colubriformis*	*T. circumcincta*	*T. vitrinus*	*C. oncophora*	Mixed Infection
**2%**	91.25 (4.82) ^a^	98.80 (0.97) ^a^	94.65 (2.28) ^a^	100 ^a^	100 ^a^	24.58 (3.62) ^b^	84.88 (12.14) ^a^
**1%**	87.36 (4.46) ^a^	98.71 (1.05) ^a^	81.11 (4.80) ^a^	100 ^a^	99 (0.76) ^a^	15.80 (2.69) ^b^	80.35 (13.28) ^a^
**0.5%**	67.77 (4.03) ^b^	95.65 (3.55) ^a^	49.79 (4.02) ^b^	99 (0.82) ^a^	92 (0.64) ^a^	6.40 (2.71) ^b^	68.44 (14.64) ^a^
**0.25%**	38 (3.89) ^b^	69 (8.35) ^b^	12.55 (5.23) ^b^	95.95 (1.73) ^a^	61.1 (4.53) ^b^	2.22 (1.81) ^b^	46.50 (14.55) ^b^
**0.125%**	29.14 (3.31) ^b^	42.21 (9.04) ^b^	2.22 (1.81) ^b^	65.33 (6.42) ^b^	15 (1.80) ^b^	0.00 ^b^	25.67 (10.30) ^b^
**IC_50_**	0.26	0.15	0.48	0.11	0.21	3.31	0.22
**R*^2^***	0.94	0.93	0.98	0.98	0.99	0.88	0.75
**+ve control**	87.18 (3.17) ^a^	87.80 (1.85) ^a^	91 (1.46) ^a^	85 (2.36) ^a^	93.27 (0.34) ^a^	94.55 (2.23) ^a^	89.81 (1.53) ^a^
**–ve control**	0.00 ^b^	0.00 ^b^	0.00 ^b^	0.00 ^b^	0.00 ^b^	00.00 ^b^	00.00 ^b^

Data are presented as mean percentage of larval motility inhibition (± SEM). ^a^ No statistical difference between positive control and treatments (*p* > 0.05). ^b^ Significant difference between positive control and treatments (*p* < 0.05; ANOVA followed by Tukey test). Abbreviations: F.C. = Final concentration; +ve control (positive control; L3s treated with 20 mg/mL levamisole); –ve control (negative control; L3s treated with PBS-Tween 2%); IC_50_ = inhibitory concentration 50%; R^2^ = nonlinear correlation.

**Table 2:** Fluorescence intensity of Sytox green of L3s treated with a combined coriander oil-linalool preparation at five different concentrations compared with positive control (dead L3s), negative control (live L3s) and blank (L3s-free).

F.C. (%)	*H. contortus*	*T. axei*	*T. colubriformis*	*T. circumcincta*	*T. vitrinus*	*C. oncophora*	Mixed Infection
**2%**	100 ^a^	100 ^a^	100 ^a^	100 ^a^	100 ^a^	76.1 (8.5) ^b^	100 ^a^
**1%**	69.2 (13.4) ^a^	100 ^a^	91.6 (3.33) ^a^	100 ^a^	88.8 (6.6) ^a^	76.1 (4.2) ^b^	85.7 (7.1) ^a^
**0.5%**	46.1 (11.5) ^b^	88.8 (6.6) ^a^	83.3 (8.33) ^a^	81.8 (7.2) ^a^	55.5 (8.8) ^b^	73.9 (2.8) ^b^	71.4 (1.43) ^b^
**0.25%**	42.3 (11.5) ^b^	77.7 (13.1) ^b^	58.3 (5.2) ^b^	72.7 (8.1) ^a^	55.5 (5.5) ^b^	65.2 (4.5) ^b^	57.1 (2.49) ^b^
**0.125%**	19.2 (1.9) ^b^	44.4 (16.3) ^b^	58.3 (1.6) ^b^	27.2 (9.1) ^b^	22.2 (3.3) ^b^	28.5 (4.8) ^b^	35.7 (7.4) ^b^
**IC_50_ (%)**	0.58	0.14	0.104	0.18	0.21	0.20	0.23
**R^2^**	0.38	0.17	0.22	0.66	0.12	0.21	0.79
**+ve control**	92.3 (7.3) ^a^	88.7 (5.4) ^a^	83.3 (4.6) ^a^	72.7 (9.1) ^a^	88.8 (7.3) ^a^	100 ^a^	100 ^a^
**–ve control**	34.6 (3.8) ^b^	22.2 (5.7) ^b^	33.3 (8.3) ^b^	27.2 (7.2) ^b^	33.3 (3.4) ^b^	32.4 (2.7) ^b^	34.6 (1.3) ^b^

Data are presented as the normalized mean of fluorescence intensity (± SEM) and show the effect of treatment with coriander oil-linalool combination on the fluorescence uptake of Sytox green by dead L3s. ^a^ No statistical difference between positive control and treatments (*p* > 0.05). ^b^ Significant difference between positive control and treatments (*p* < 0.05; ANOVA followed by Tukey test). Abbreviations: F.C. = Final concentration; +ve control (positive control; L3s treated with 50% methanol); –ve control (negative control; L3s treated with PBS-Tween 2%); IC_50_ = inhibitory concentration 50%; R^2^ = nonlinear correlation.

**Table 3:** Fluorescence intensity of propidium iodide of L3s treated with a combined coriander oil-linalool preparation at five different concentrations compared with positive control (dead L3s), negative control (live L3s) and blank (L3s-free).

F.C. (%)	*H. contortus*	*T. axei*	*T. colubriformis*	*T. circumcincta*	*T. vitrinus*	*C. oncophora*	Mixed Infection
**2%**	100 ^a^	100 ^a^	100 ^a^	100 ^a^	100 ^a^	78.26 (5.9) ^b^	100 ^a^
**1%**	56.1 (6.8) ^b^	76.4 (4.7) ^a^	76.3 (3.8) ^a^	100 ^a^	84.6 (3.3) ^a^	72.46 (3.3) ^b^	73.0 (8.4) ^a^
**0.5%**	39 (5.6) ^b^	58.8 (7.5) ^b^	34.2 (13.8) ^b^	40 (9.2) ^b^	46.1 (8.5) ^b^	69.5 (3.3) ^b^	42.3 (5.1) ^b^
**0.25%**	39 (10.8) ^b^	58.8 (2.9) ^b^	31.5 (9.05) ^b^	40 (8.8) ^b^	34.6 (8.5) ^b^	55.1 (8.8) ^b^	34.6 (5.1) ^b^
**0.125%**	2.4 (0.1) ^b^	41.1 (4.5) ^b^	15.7 (5.8) ^b^	6.6 (1.9) ^b^	30.7 (1.4) ^b^	50.7 (7.3) ^b^	11.5 (6.7) ^b^
**IC_50_ (%)**	0.58	0.25	0.52	0.41	0.38	0.42	0.49
**R^2^**	0.37	0.07	0.35	0.12	0.14	0.09	0.75
**+ve control**	69.8 (3.2) ^a^	97.7 (1.17) ^a^	80.9 (8.5) ^a^	94.2 (2.6) ^a^	88.8 (4.07) ^a^	100 ^a^	89.8 (6.7) ^a^
**–ve control**	50.6 (4.3) ^b^	62.2 (7.5) ^b^	39.6 (3.1) ^b^	71.1 (3.4) ^b^	51.8 (1.8) ^b^	50.7 (7.2) ^b^	55.9 (5.1) ^b^

Data are presented as the normalized mean of fluorescence intensity (± SEM) and show the effect of exposure to coriander oil-linalool combination on the fluorescence uptake of propidium iodide by dead L3s. ^a^ Non-statistical difference between positive control and treatments (*p* > 0.05) ^b^ Significant difference between positive control and treatments (*p* < 0.05; ANOVA followed by Tukey test) Abbreviations: F.C. = Final concentration; +ve control (positive control; L3s treated with 50% methanol); –ve control (negative control; L3s treated with PBS-Tween 2%); IC_50_ = inhibitory concentration 50%; R^2^ = nonlinear correlation.

**Table 4:** Fluorescence intensity of C_12_-resazurin of L3s treated with a combined coriander-linalool preparation at five different concentrations compared with positive control (dead L3s), negative control (live L3s) and blank (L3s-free).

F.C. (%)	*H. contortus*	*T. axei*	*T. colubriformis*	*T. circumcincta*	*T. vitrinus*	*C. oncophora*	Mixed Infection
**2%**	0.00 ^a^	0.00 ^a^	0.00 ^a^	0.00 ^a^	0.00 ^a^	30.7 (4.16) ^a^	0.00 ^a^
**1%**	23.1 (3.2) ^a^	40 (5.7) ^a^	23.1 (7.6) ^a^	53.8 (1.5) ^b^	25 (2.0) ^a^	100 ^b^	40 (6.8) ^b^
**0.5%**	46.1 (4.52) ^b^	60 (1.1) ^b^	69.2 (2.8) ^b^	69.2 (7.5) ^b^	80 (4.0) ^b^	53.8 (6.2) ^b^	66.6 (4.5) ^b^
**0.25%**	69.2 (3.9) ^b^	70 (3.6) ^b^	100 ^b^	90 (2.5) ^b^	90 (6.1) ^b^	76.9 (8.3) ^b^	80 (4.5) ^b^
**0.125%**	100 ^b^	100 ^b^	100 ^b^	100 ^b^	100 ^b^	76.9 (12.5) ^b^	100 ^b^
**IC_50_ (%)**	0.31	0.36	0.66	0.56	0.72	0.15	0.51
**R2**	0.05	0.01	0.18	0.03	0.24	0.09	0.17
**+ve control **	30.7 (1.32) ^a^	52.6 (1.52) ^a^	48.7 (2.5) ^a^	30.7 (2) ^a^	30 (2.04) ^a^	45.8 (2.0) ^a^	26.6 (6.6) ^a^
**–ve control **	66.0 (3.7) ^b^	78.9 (5.2) ^b^	79.4 (5.3) ^b^	77.5 (5) ^b^	71.4 (4.0) ^b^	72.9 (2.0) ^b^	75 (2.2) ^b^

Data are presented as the normalized mean of fluorescence intensity (± SEM) and show the effect of coriander oil-linalool combination on the fluorescence uptake of C_12_-resazurin by live L3s. ^a^ Non-statistical difference between positive control and treatments (*p* > 0.05) ^b^ Significant difference between positive control and treatments (*p* < 0.05; ANOVA followed by Tukey test). Abbreviations: F.C. = Final concentration; +ve control (positive control; L3s treated with 50% methanol); –ve control (negative control; L3s treated with PBS-Tween 2%); IC_50_ = inhibitory concentration 50%; R^2^ = nonlinear correlation.

## Supplementary Materials

The following are available online at https://www.mdpi.com/2076-0817/9/9/740/s1, Figure S1: Combined effect of coriander oil and linalool on L3s of GINs, Figure S2: Chromatographic analysis of extracted coriander oil, Video S1: Effect of coriander oil treatment (1% concentration) on L3s of GIN.
