# Supplementary material for: Nematocidal Effects of a Coriander Essential Oil and Five Pure Principles on the Infective Larvae of Major Ovine Gastrointestinal Nematodes In Vitro
Source: Pathogens. 2020 Sep 9;9(9):740. doi: 10.3390/pathogens9090740 (PMC7558654; doi:10.3390/pathogens9090740)
Supplement: Supplementary file 1 [file pathogens-09-00740-s001.zip › Supplementary materials/Figure S2.docx]

**Figure S2:** Chromatographic study of extracted coriander oil.


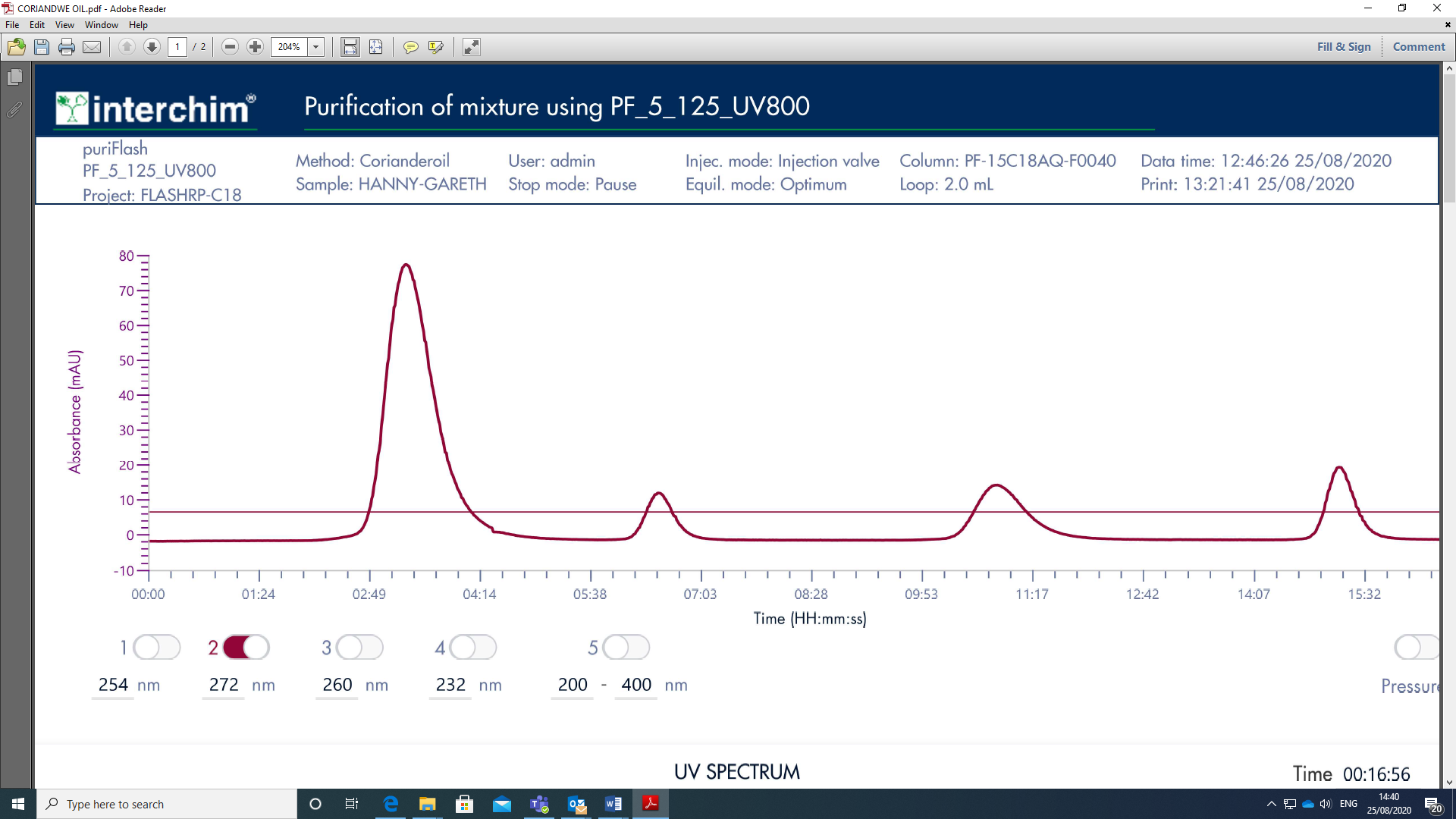


Linalool (68.03%)

Camphor (11.76%)

γ-Terpinene (10.48%)

α-Pinene (9.71%)

The chromatographic study of extracted coriander oil was conducted on a PuriFlash^®^5.125 flash system using a Puriflash C18-HP 15 micron F0040 Flash column (internal diameter 27 mm, column length of 130 mm, volume 75 mL, silica particle size: 15 um) using an isocratic mobile phase (Solvent A: Water-0.1% formic acid and 5 mM ammonium formate, Solvent B: water 10%-methanol 90%; Solvent A: Solvent B 35:65) at a flow rate of 26 mL/min and a pressure of 20 bar (290 psi). The column was equilibrated (13 min 42 sec) and calibrated with linalool, alpha-pinene, gamma-terpinene and camphor analytical standards (Sigma, UK) using a UV detection rage of 200-800 nm. The extracted coriander oil was diluted into Solvent A (1:10) and eluted on the column (injection volume 2 mL, runtime 17 min). Component concentrations were calculated using peak area calculations within the PuriFlash^®^5.125 flash software.
