# Supplementary material for: Nematocidal Effects of a Coriander Essential Oil and Five Pure Principles on the Infective Larvae of Major Ovine Gastrointestinal Nematodes In Vitro
Source: Pathogens. 2020 Sep 9;9(9):740. doi: 10.3390/pathogens9090740 (PMC7558654; doi:10.3390/pathogens9090740)

Dual Effect of Coriander and Linalool on third larval stages of gastrointestinal nematodes

Haemonchus contortus L3s after treatment with Coriander and linalool 2% (10X magnification). Dead L3s are immobile upon prodding

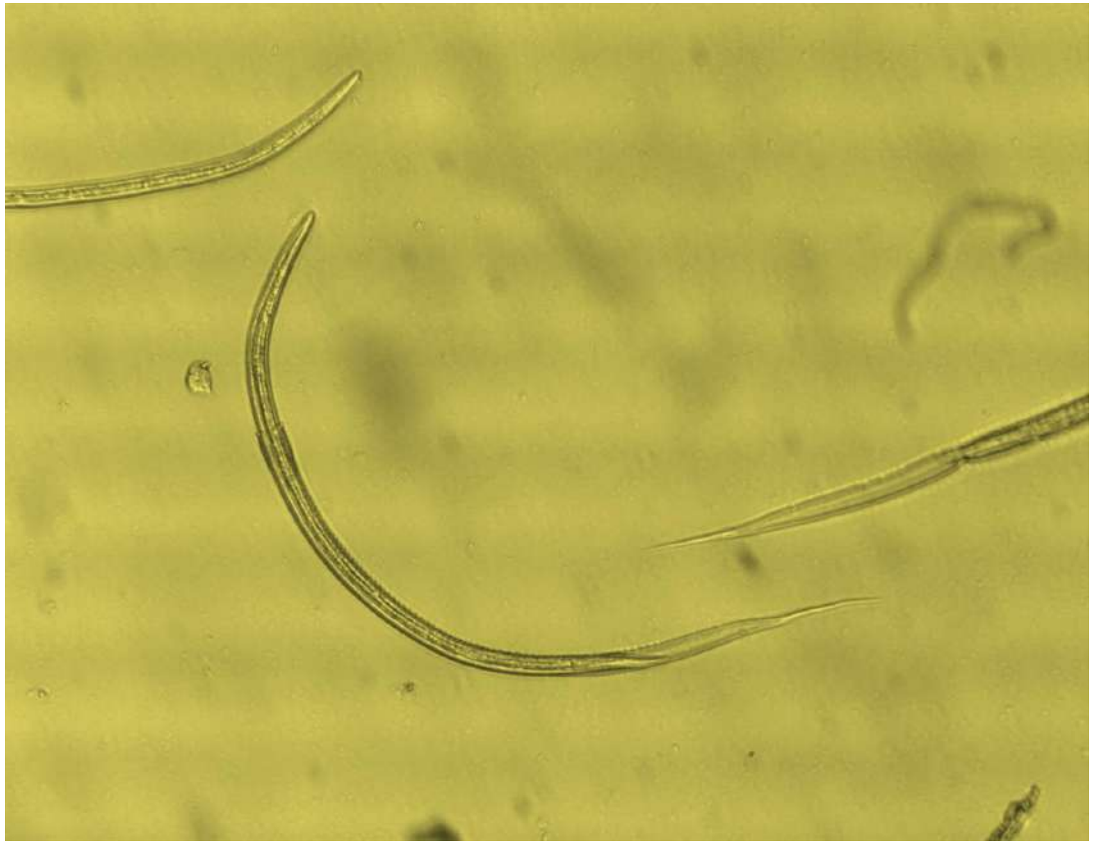

Haemonchus contortus L3s after treatment with Coriander and linalool 0.125% (10X magnification). Alive L3s are motile upon prodding

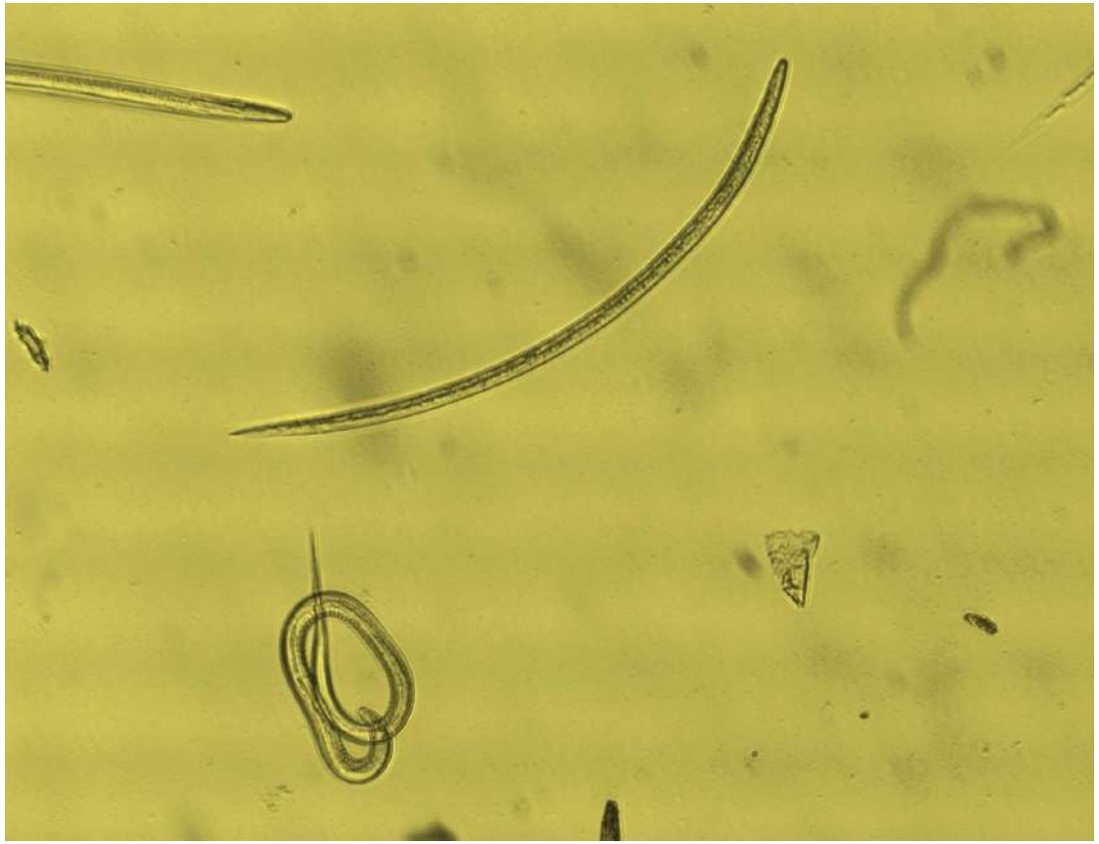

Haemonchus contortus L3s after treatment with levamisole 20 mg/ml as positive control 1 (10X magnification).

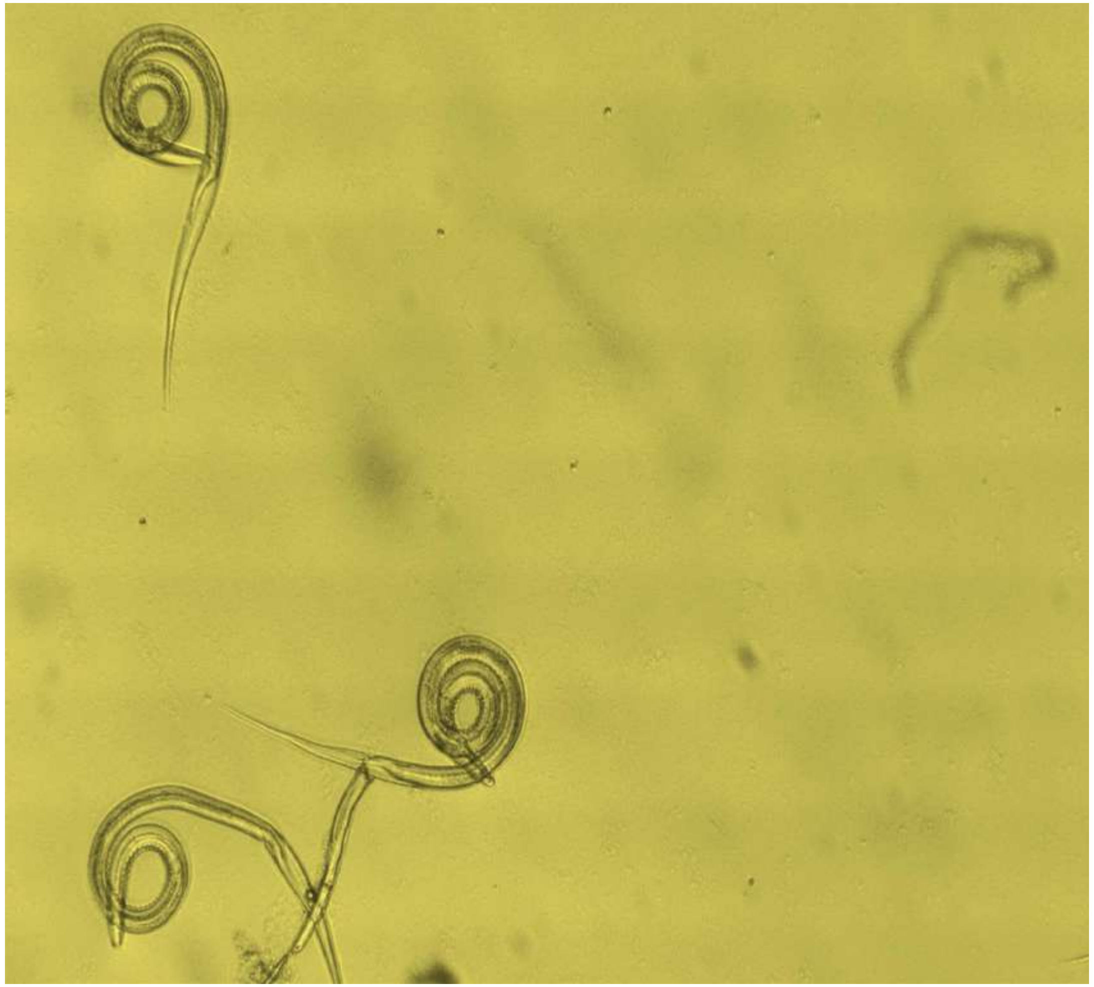

Haemonchus contortus L3s after heating treatment at 70 C for 10 minutes as positive control 2 (10X magnification). The dead Dead L3s are immobile upon prodding.

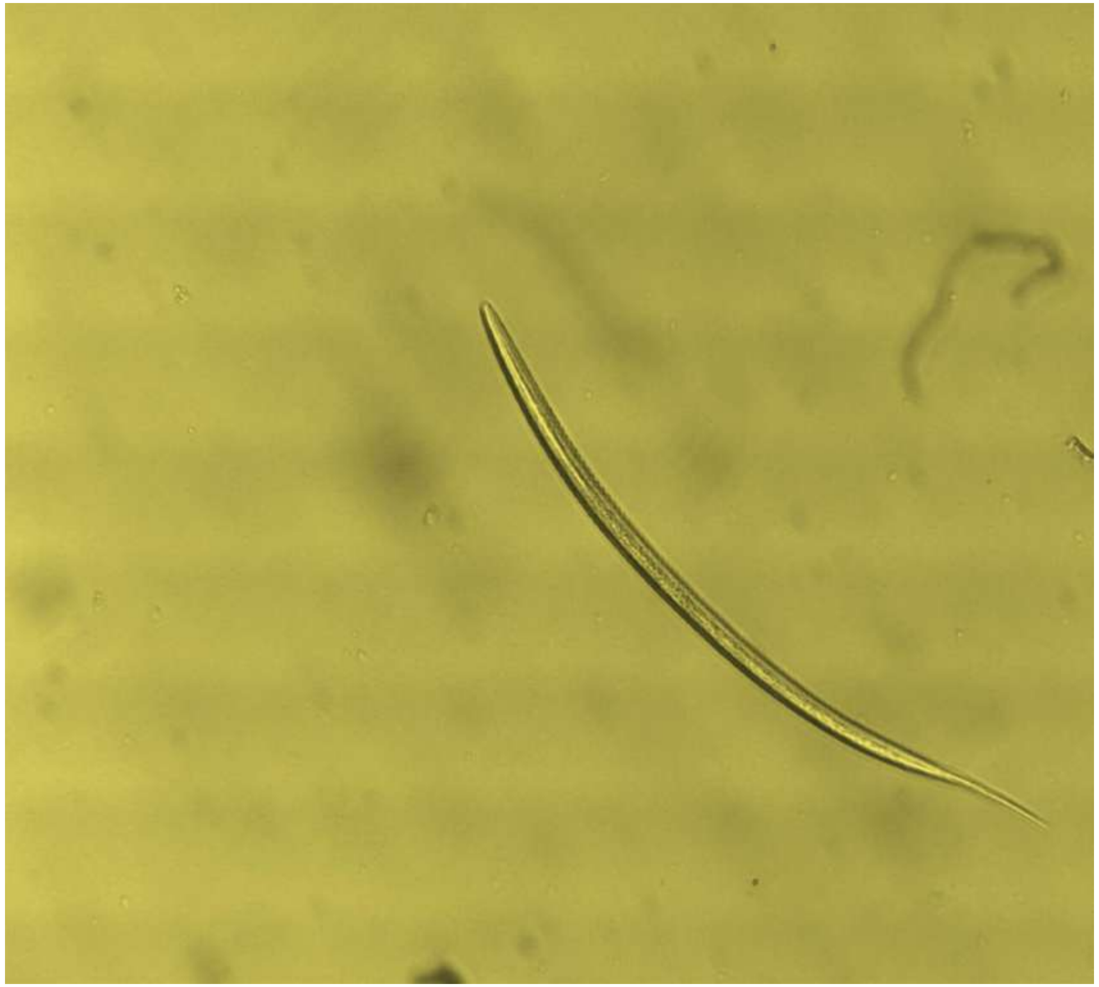

Haemonchus contortus L3s without treatment neither by Eos nor by anthelmintic drugs as negative control (10X magnification).

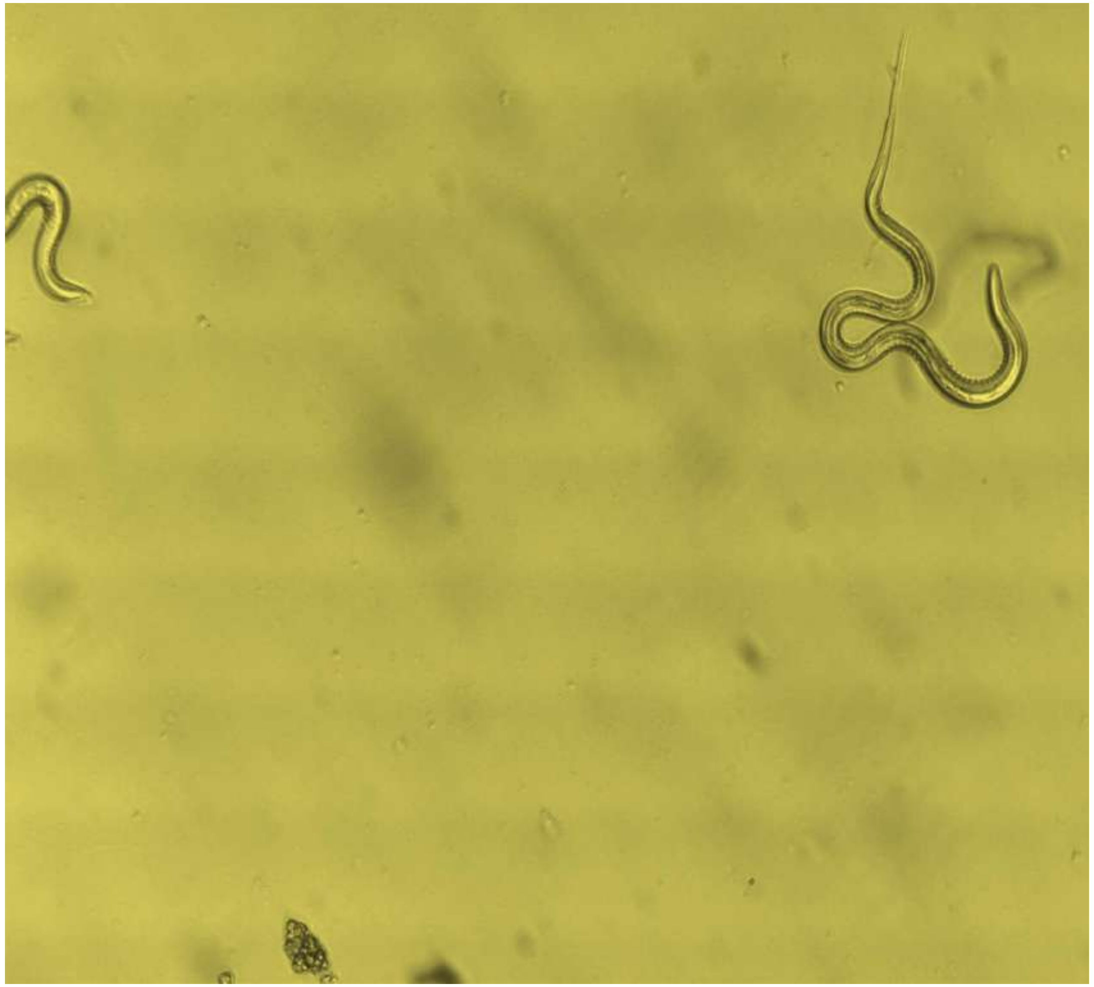

Haemonchus contortus L3s without treatment neither by Eos nor by anthelmintic drugs as negative control (25X magnification).

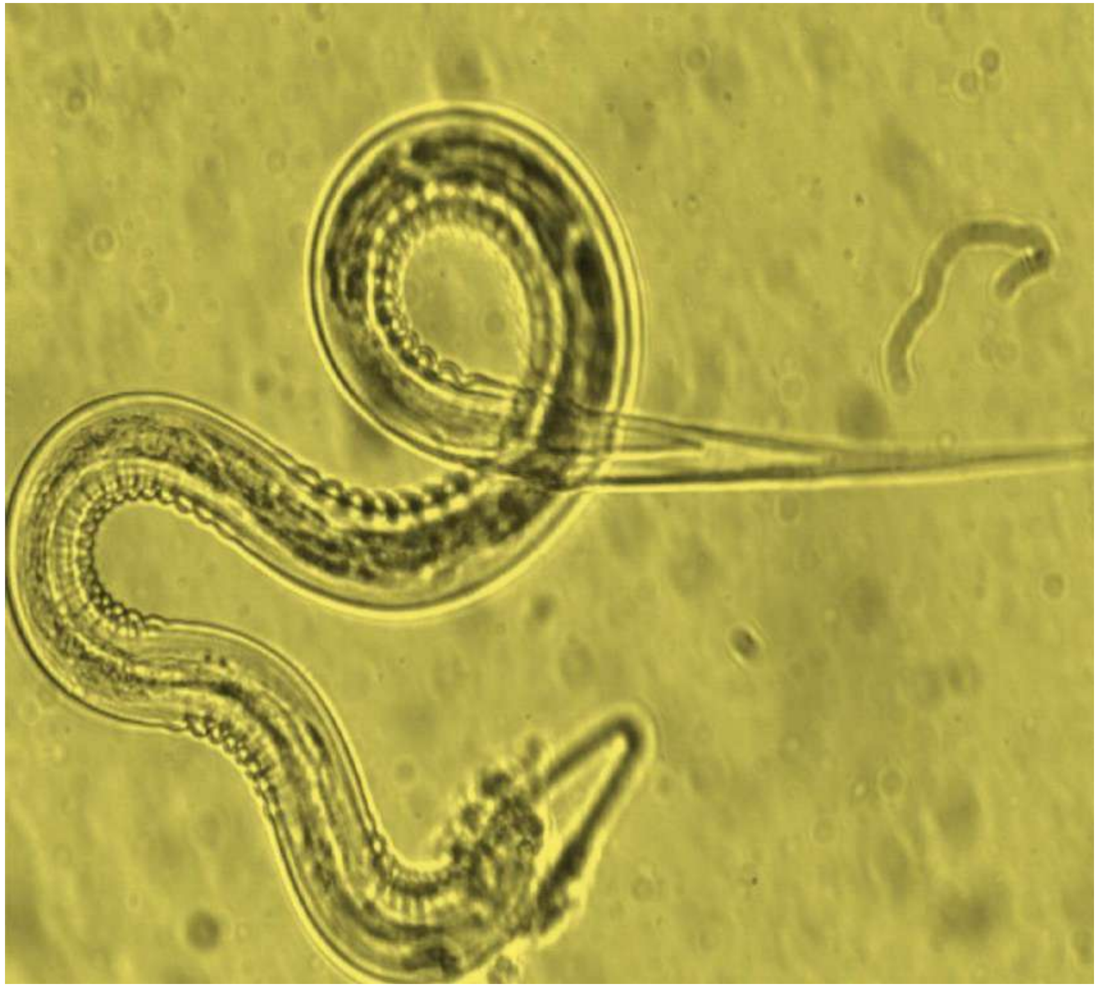

Trichostrongylus axei L3s after treatment with Coriander and linalool 2% (10X magnification). Dead L3s are immobile upon prodding

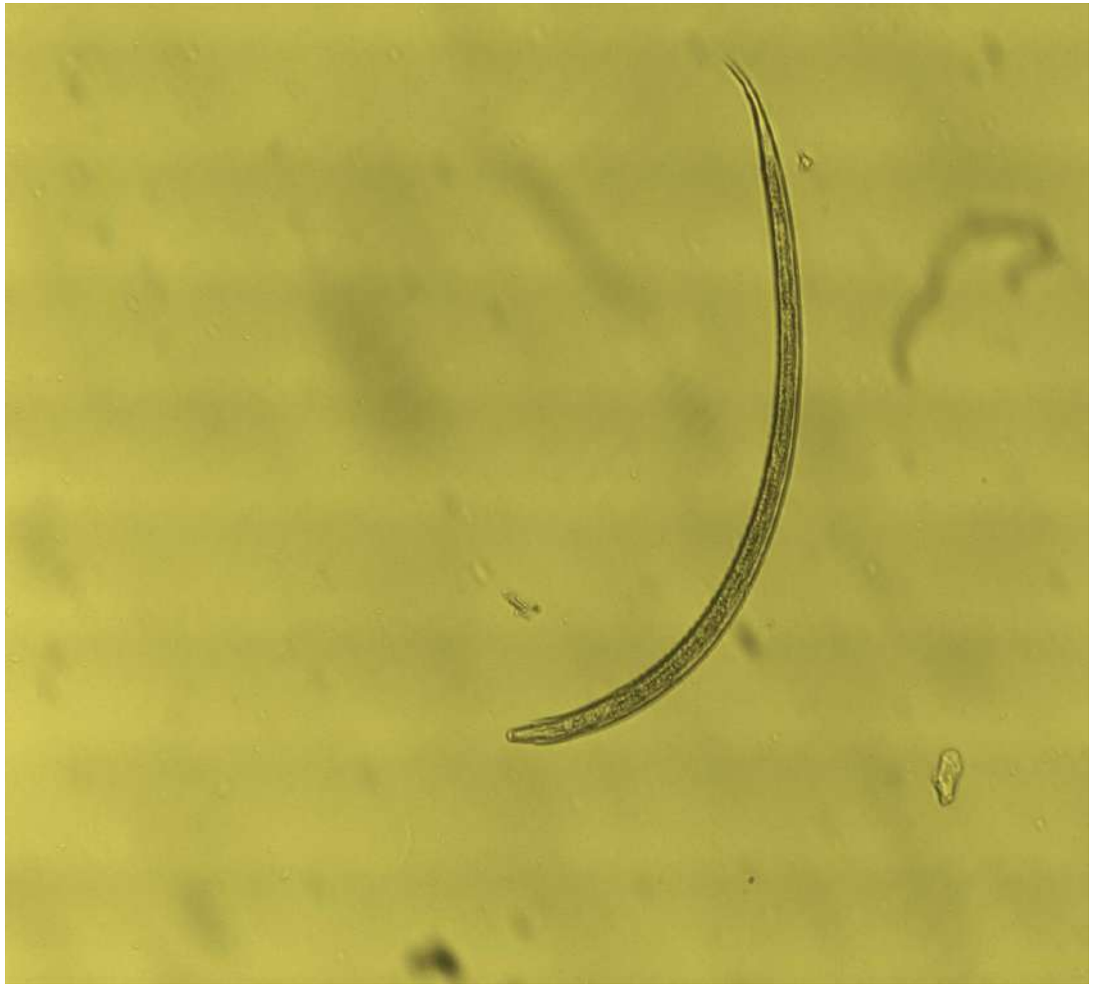

Trichostrongylus axei L3s after treatment with Coriander and linalool 0.125% (10X magnification). Alive L3s are motile upon prodding

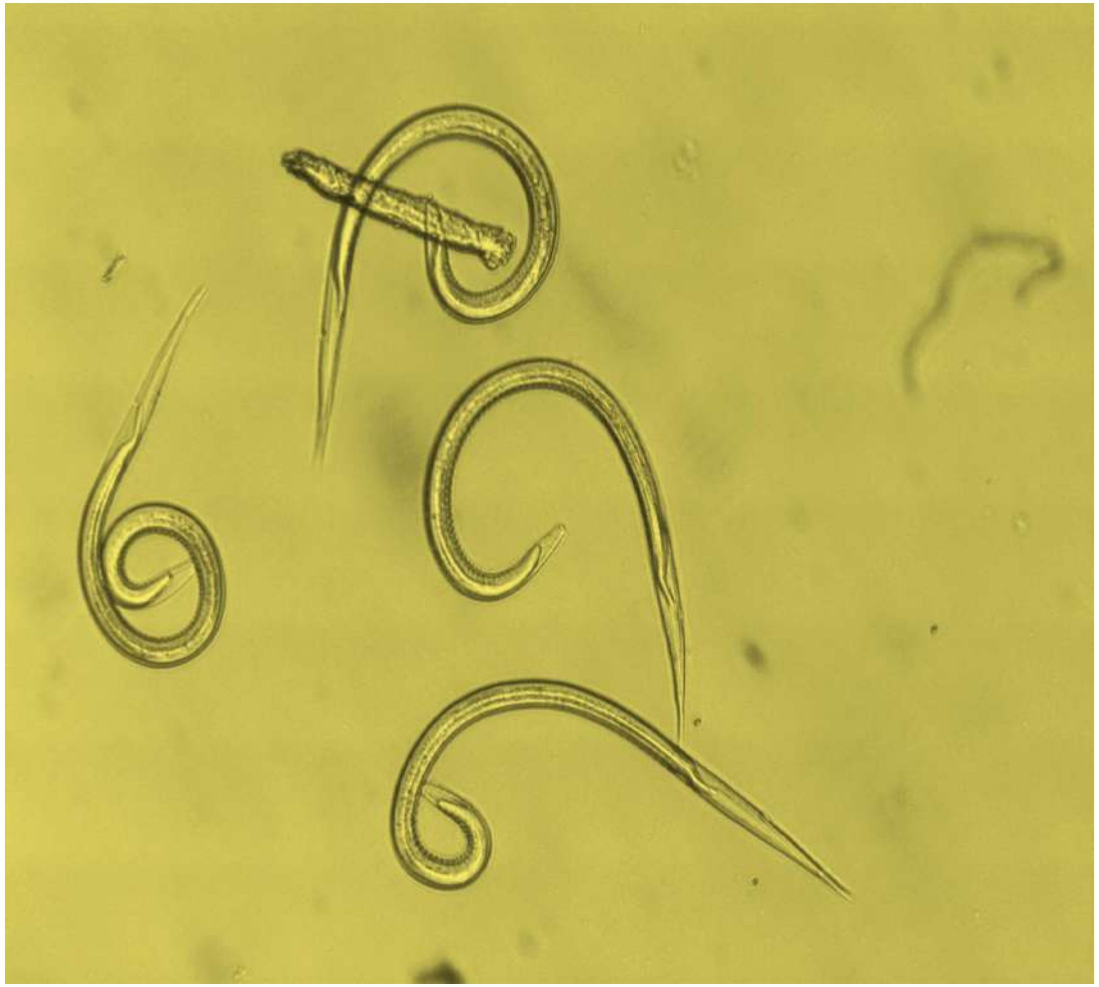

Trichostrongylus axei L3s after treatment with levamisole 20 mg/ml as positive control 1 (10X magnification).

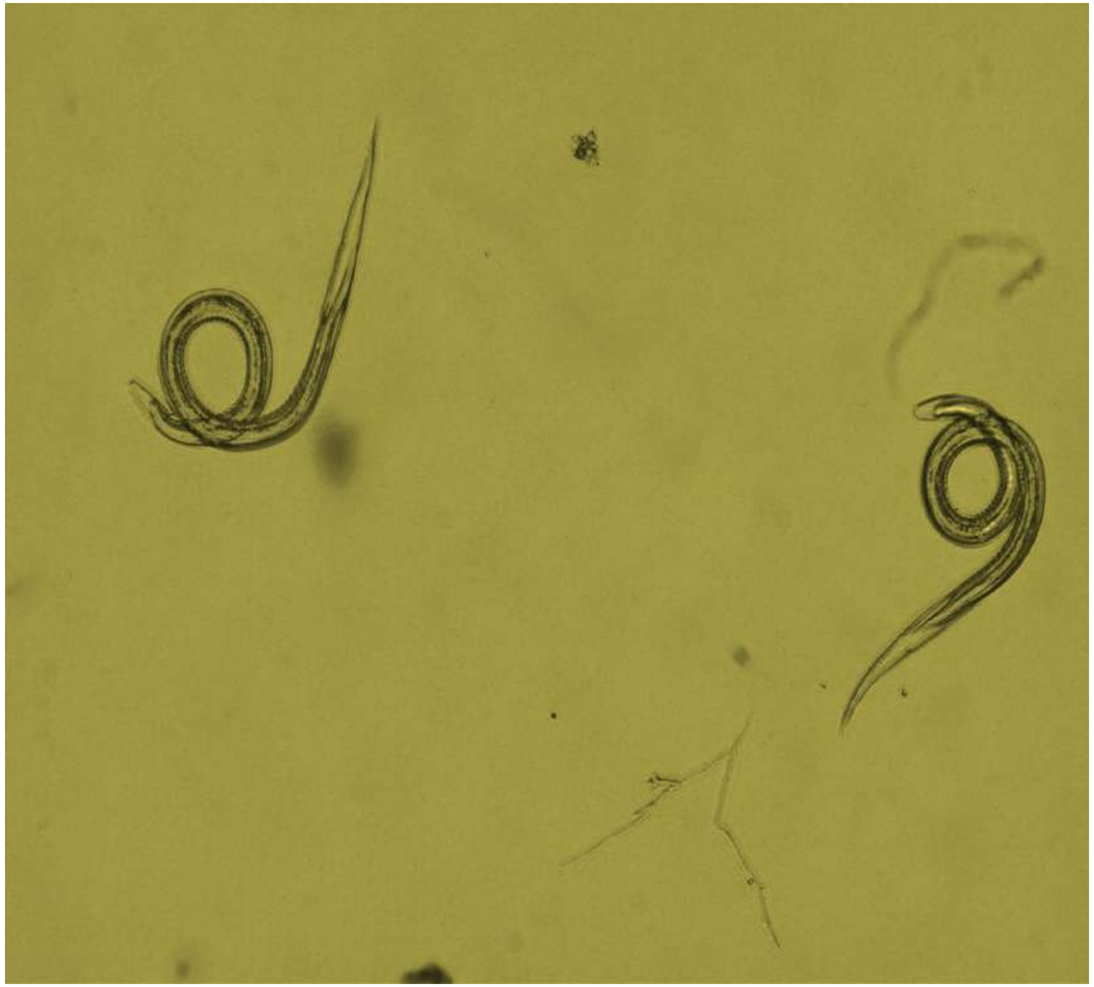

Trichostrongylus axei L3s after heating treatment at 70 C for 10 minutes as positive control 2 (10X magnification). The dead Dead L3s are immobile upon prodding.

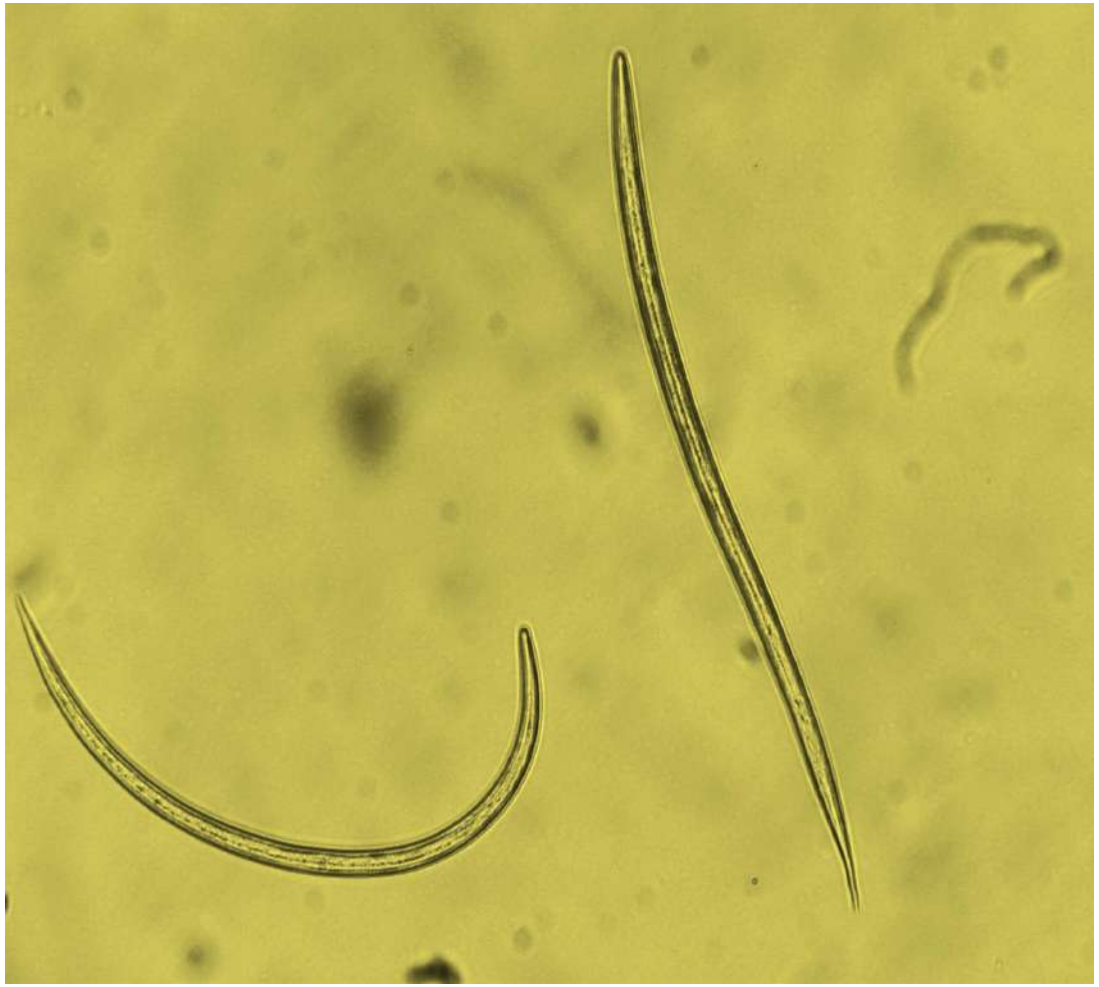

Trichostrongylus axei L3s without treatment neither by Eos nor by anthelmintic drugs as negative control (10X magnification).

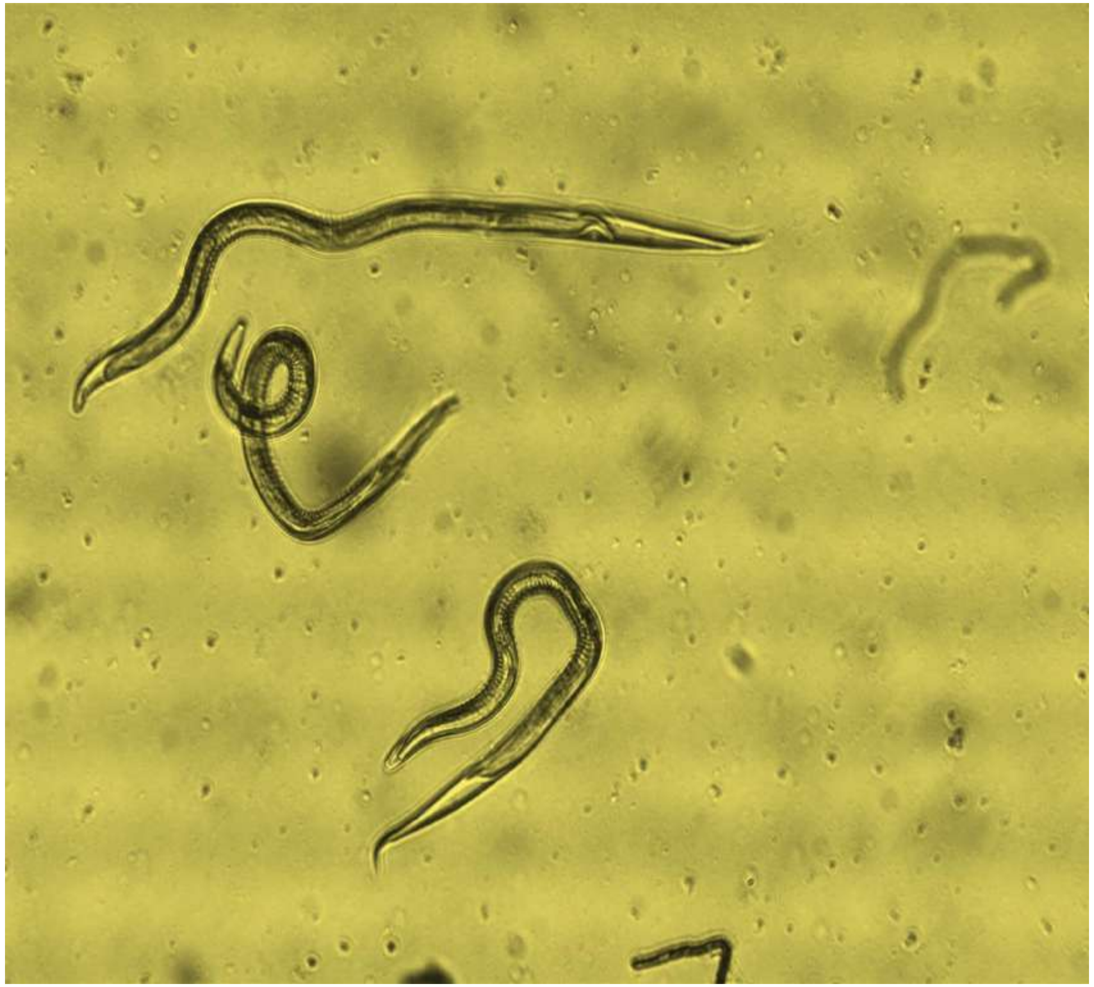

Trichostrongylus axei L3s without treatment neither by Eos nor by anthelmintic drugs as negative control (25X magnification).

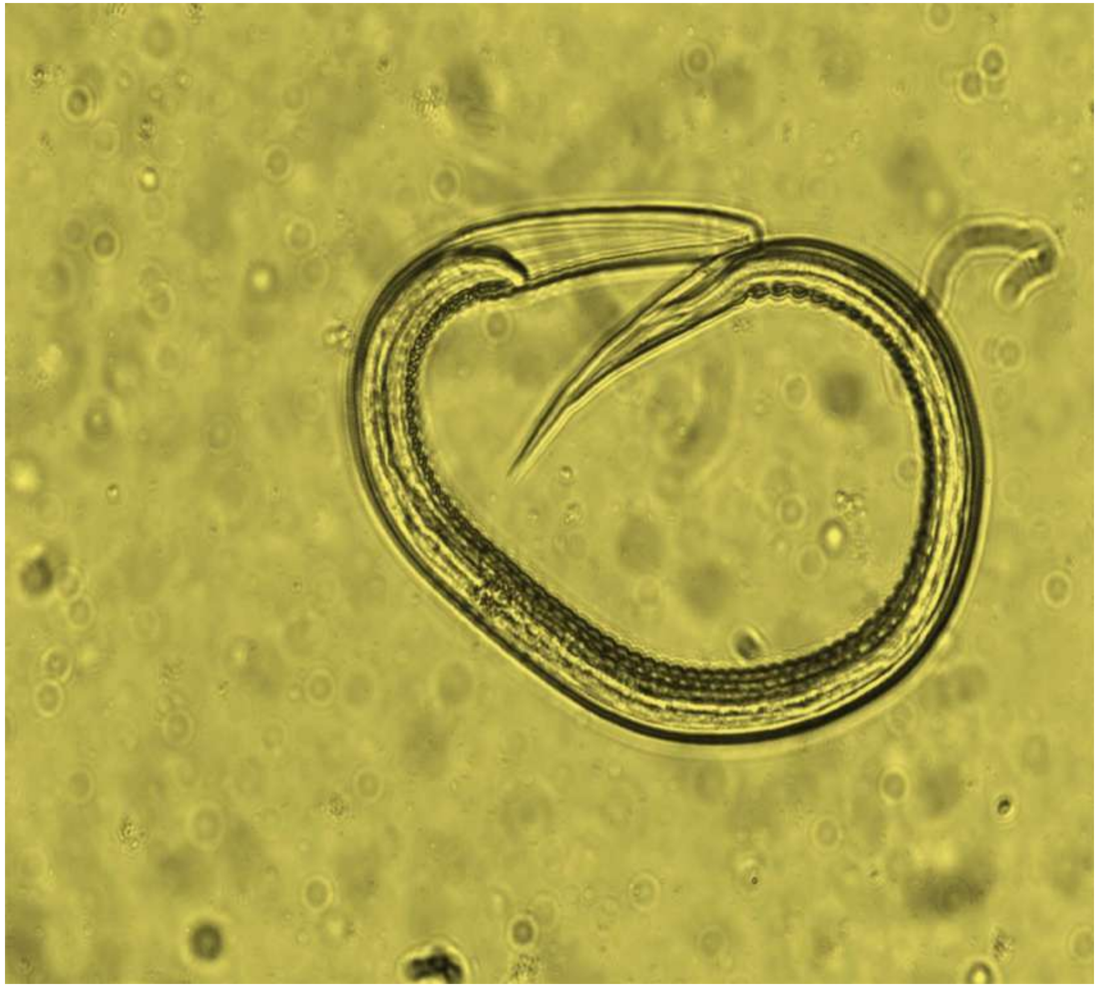

Trichostrongylus colubriformis L3s after treatment with Coriander and linalool 2% (10X magnification). Dead L3s are immobile upon prodding

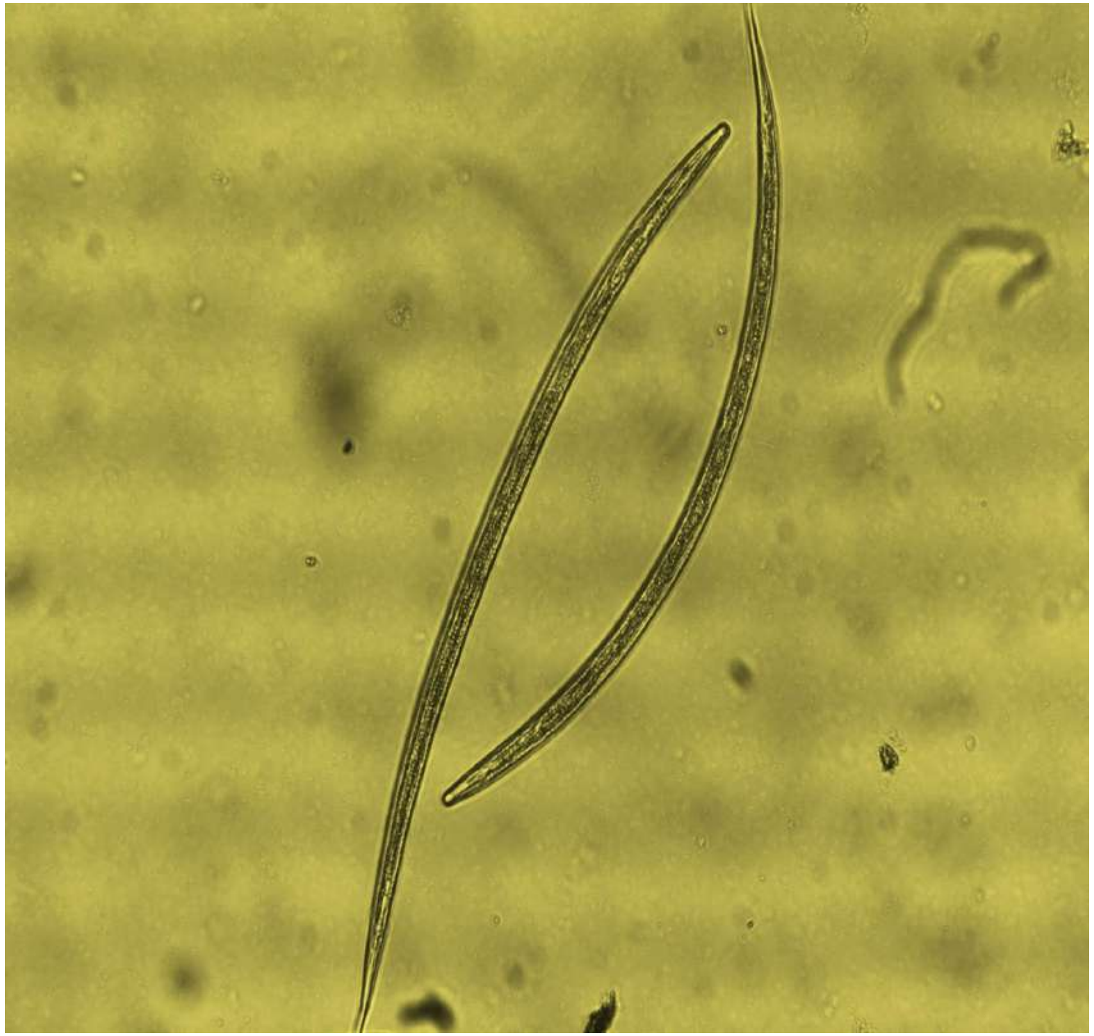

Trichostrongylus colubriformis L3s after treatment with Coriander and linalool 0.125% (10X magnification). Alive L3s are motile upon prodding

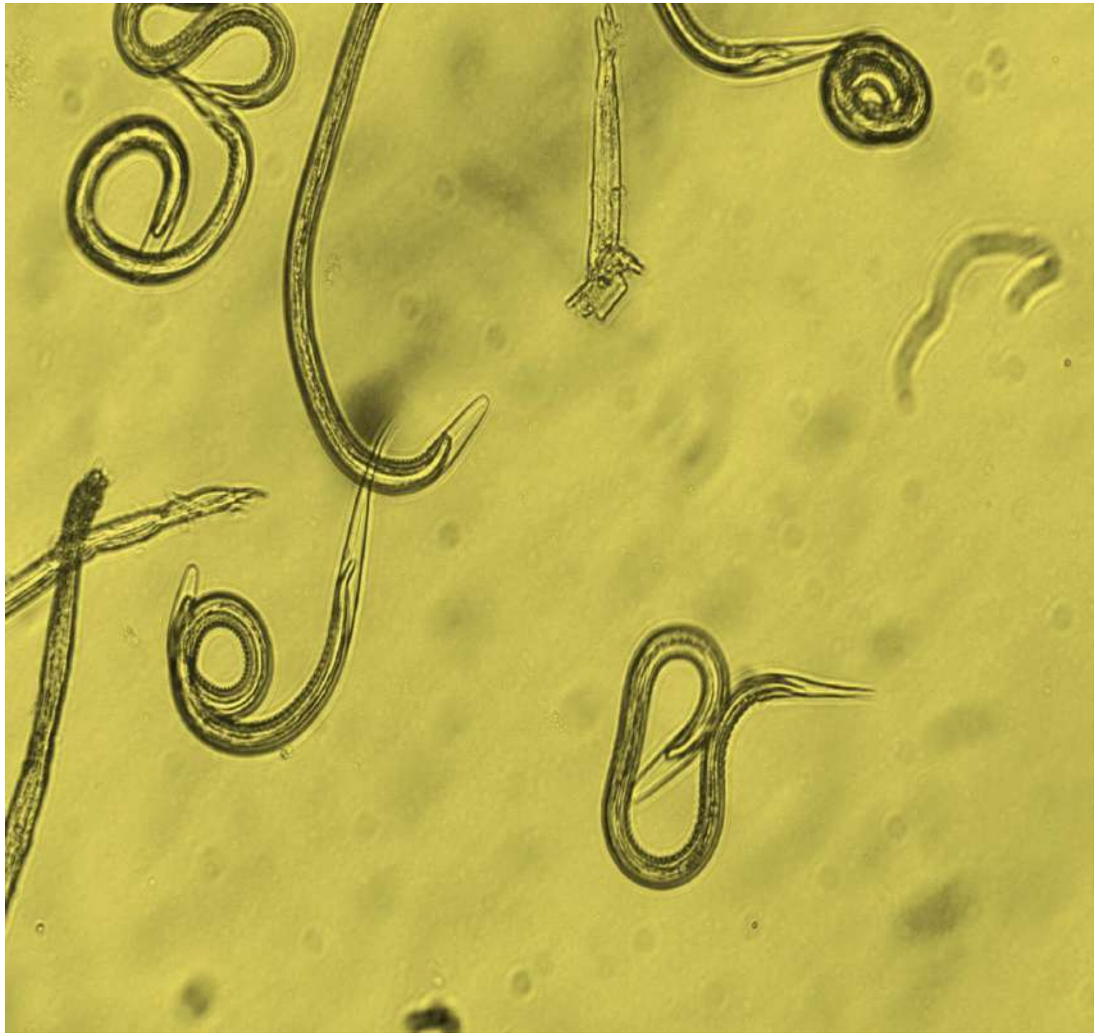

Trichostrongylus colubriformis L3s after treatment with levamisole 20 mg/ml as positive control 1 (10X magnification).

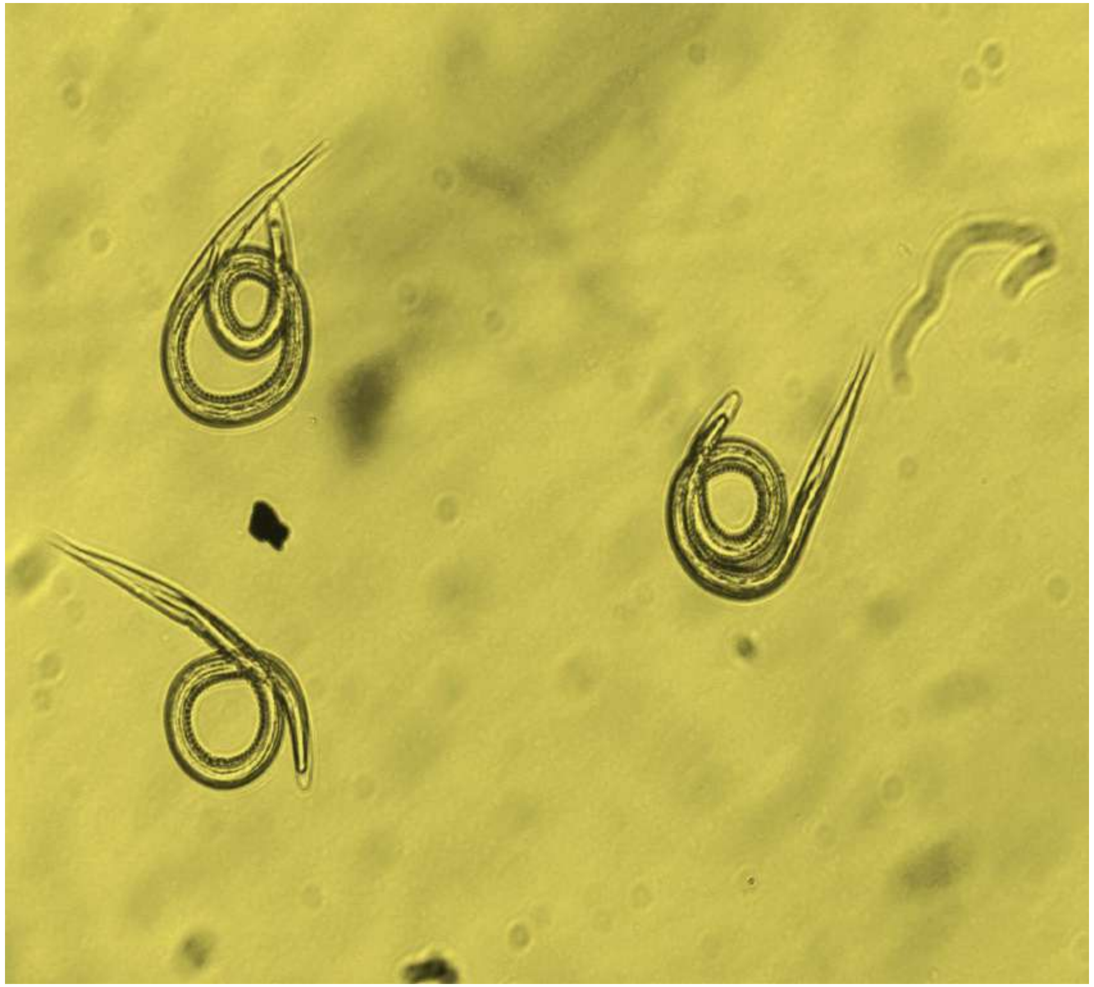

Trichostrongylus colubriformis L3s after heating treatment at 70 C for 10 minutes as positive control 2 (10X magnification). The dead Dead L3s are immobile upon prodding.

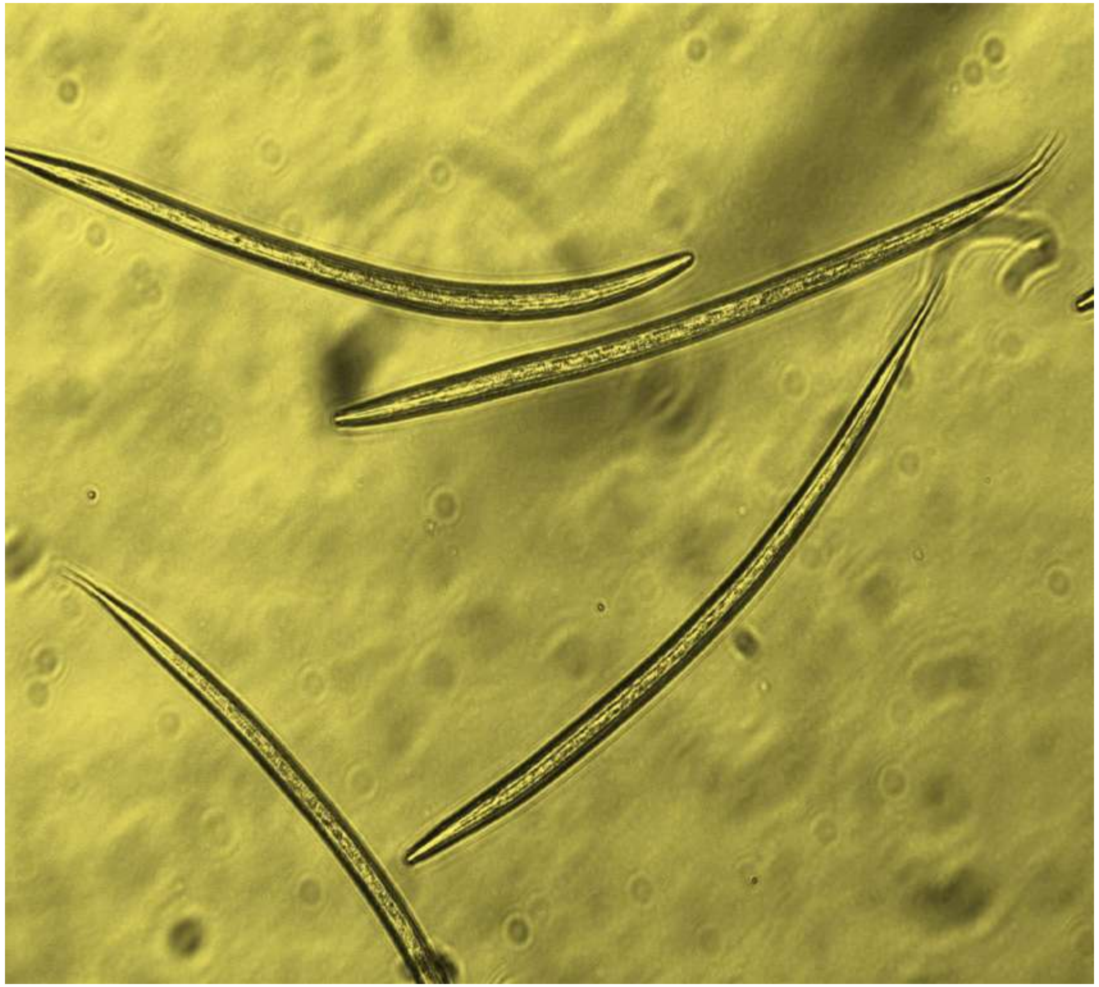

Trichostrongylus colubriformis L3s without treatment neither by Eos nor by anthelmintic drugs as negative control (10X magnification).

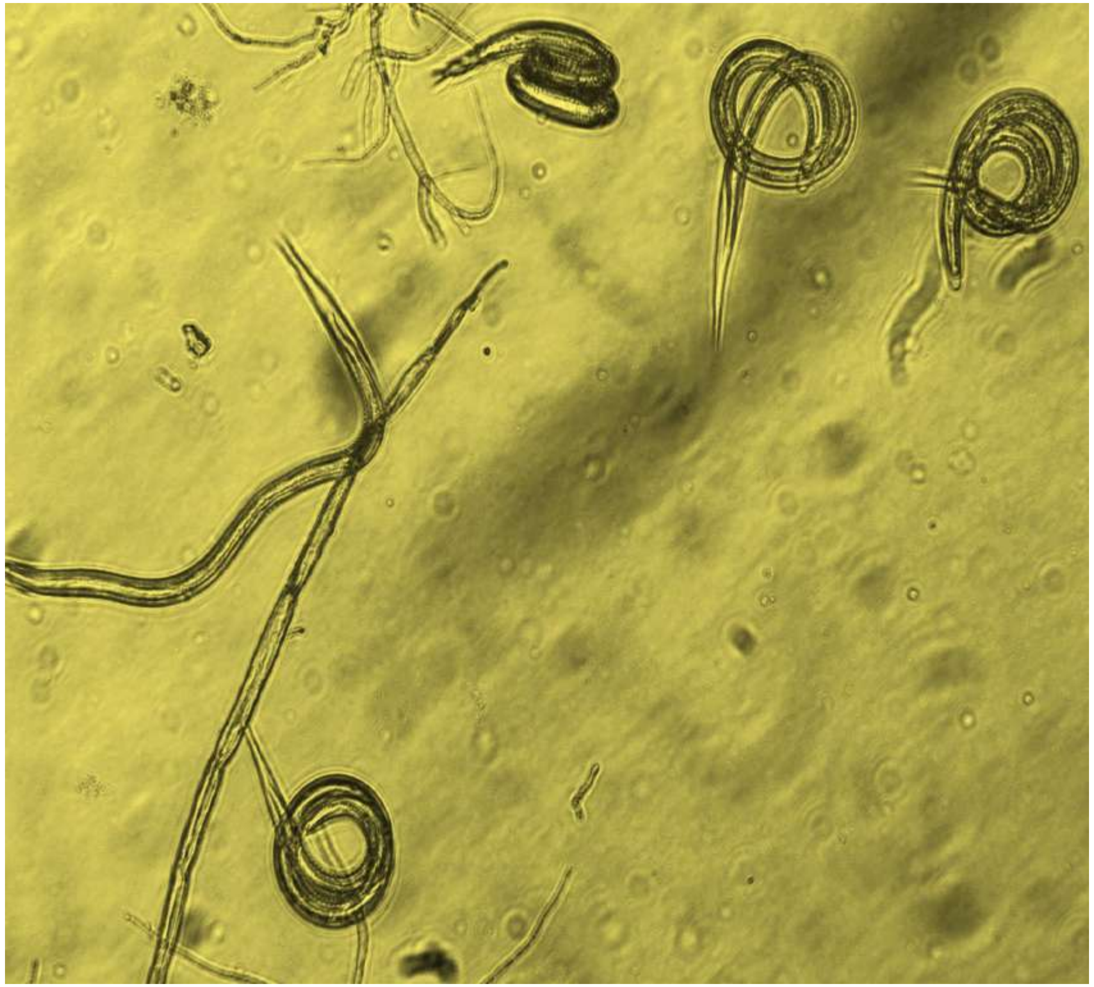

Trichostrongylus colubriformis L3s without treatment neither by Eos nor by anthelmintic drugs as negative control (25X magnification).

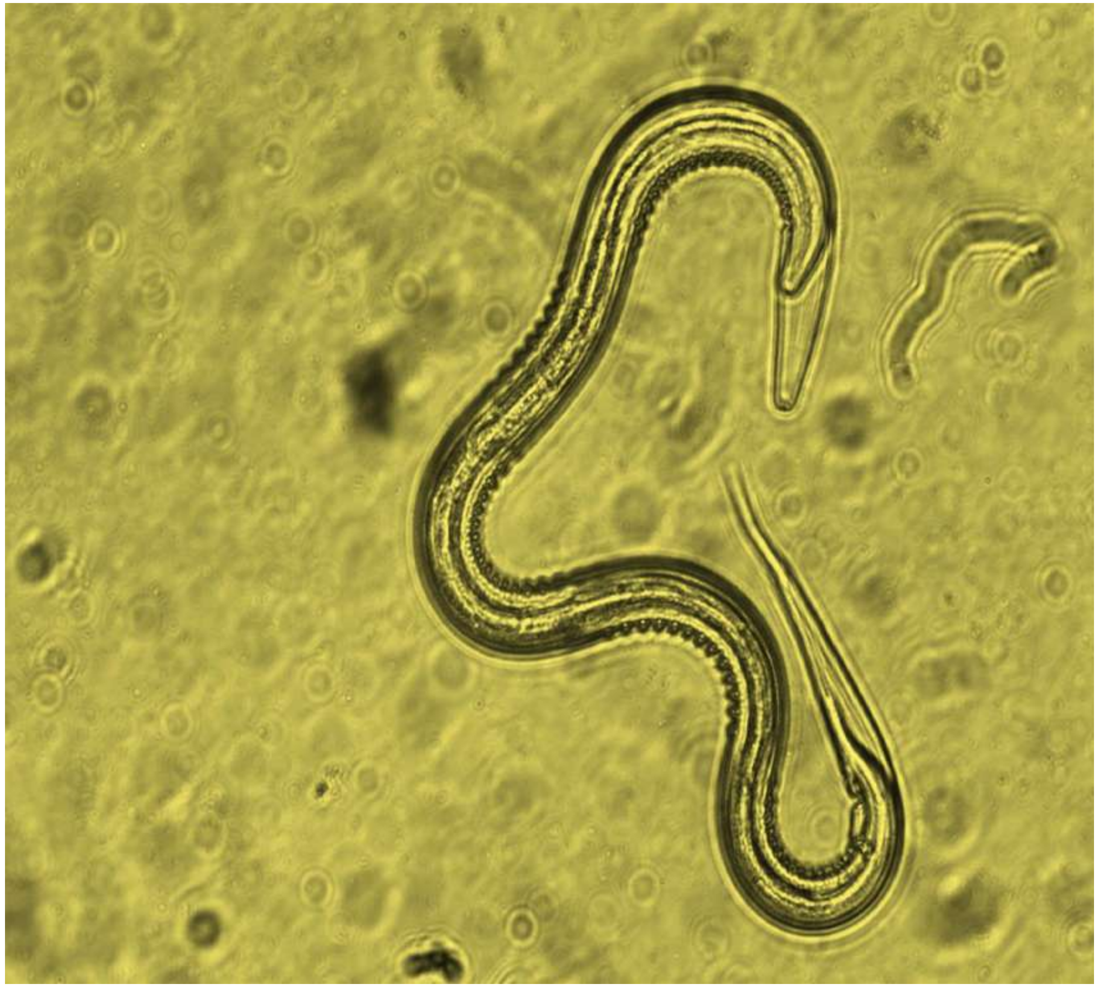

Teladorsagia circumcincta L3s after treatment with Coriander and linalool 2% (10X magnification). Dead L3s are immobile upon prodding

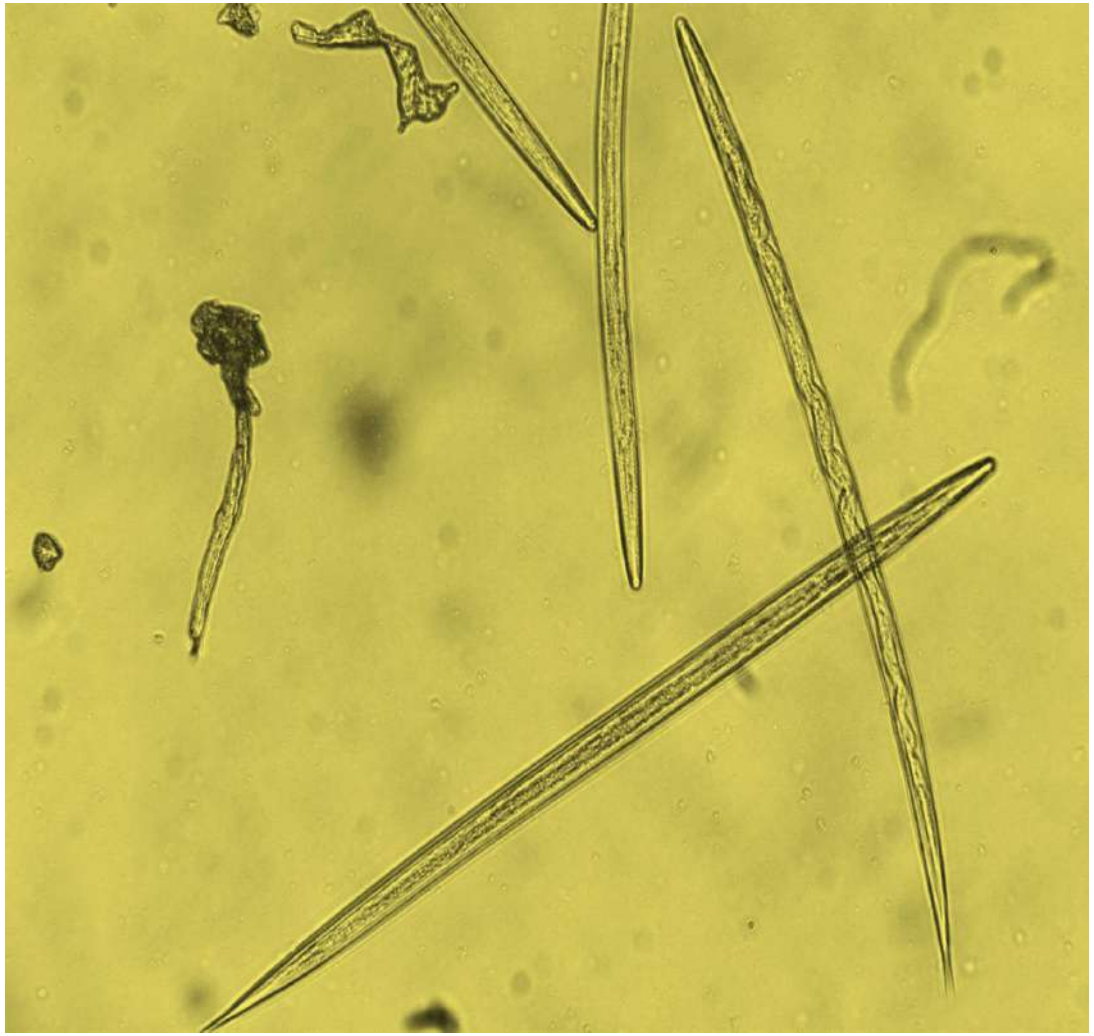

Teladorsagia circumcincta L3s after treatment with Coriander and linalool 0.125% (10X magnification). Alive L3s are motile upon prodding

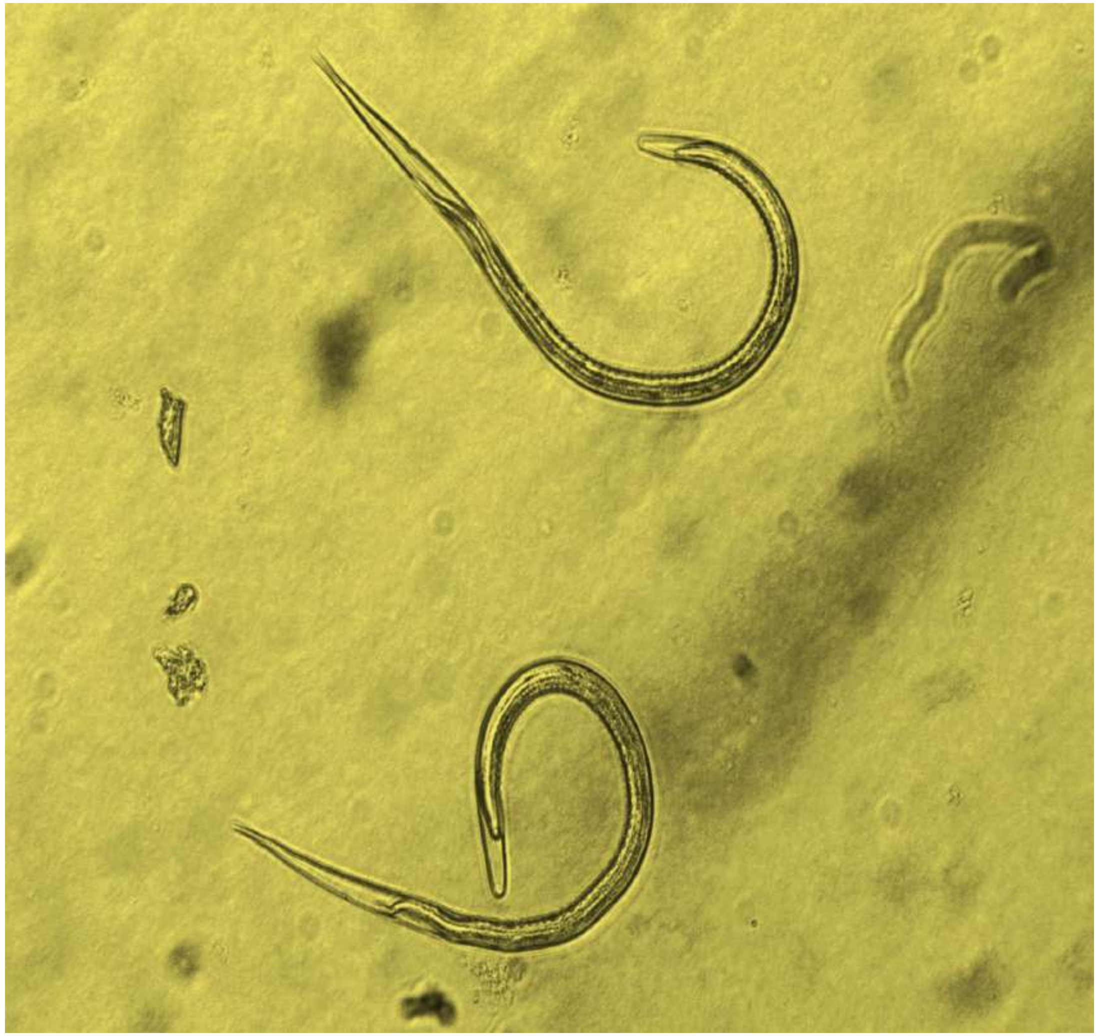

Teladorsagia circumcincta L3s after treatment with levamisole 20 mg/ml as positive control 1 (10X magnification).

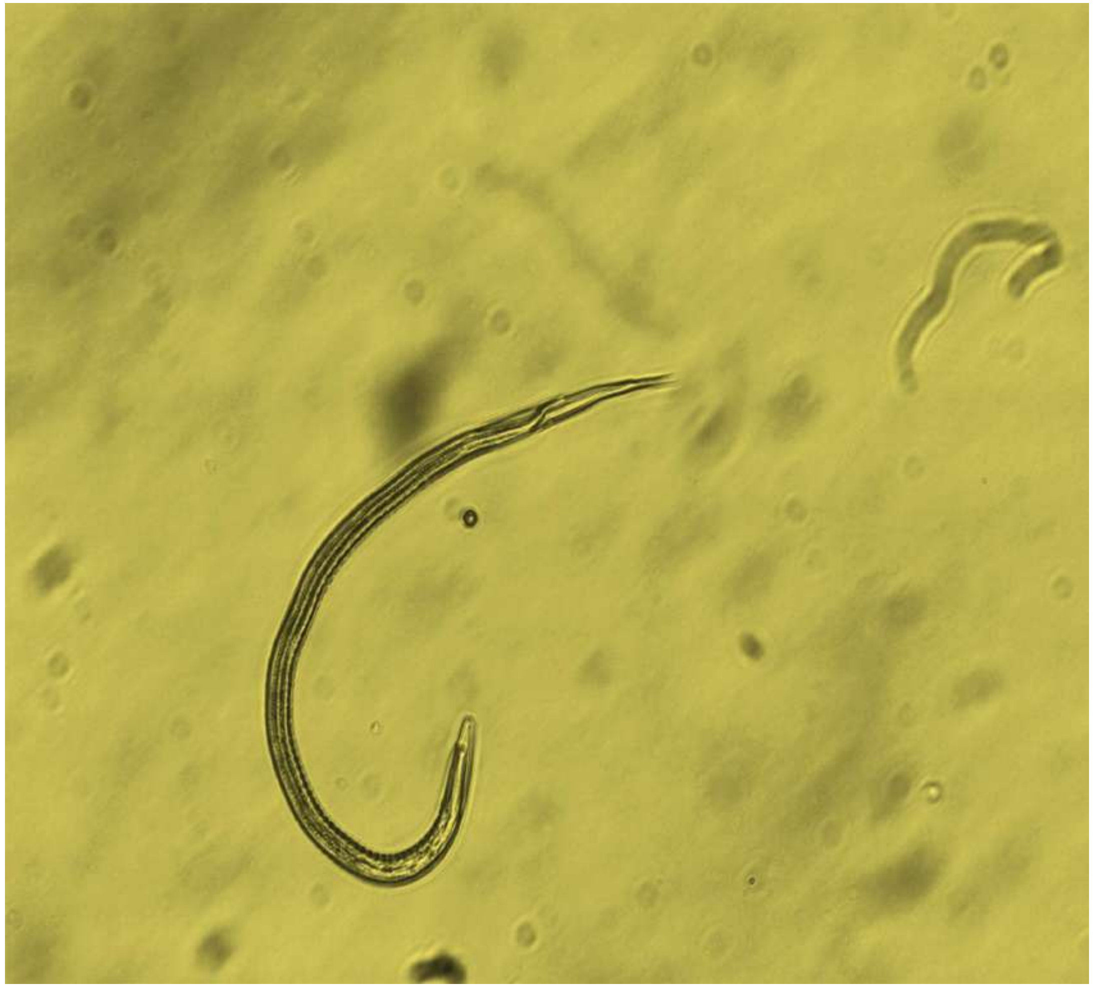

Teladorsagia circumcincta L3s after heating treatment at 70 C for 10 minutes as positive control 2 (10X magnification). The dead Dead L3s are immobile upon prodding.

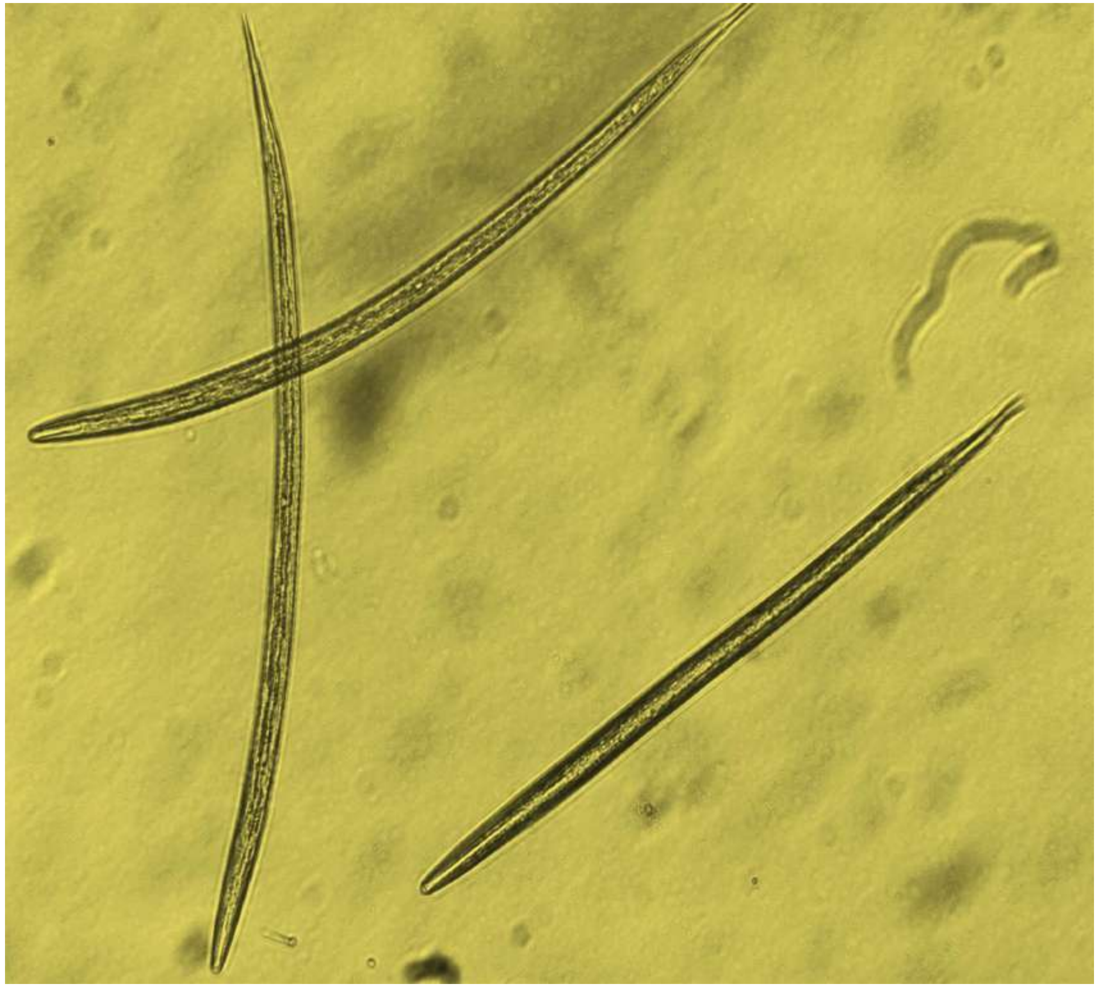

Teladorsagia circumcincta L3s without treatment neither by Eos nor by anthelmintic drugs as negative control (10X magnification).

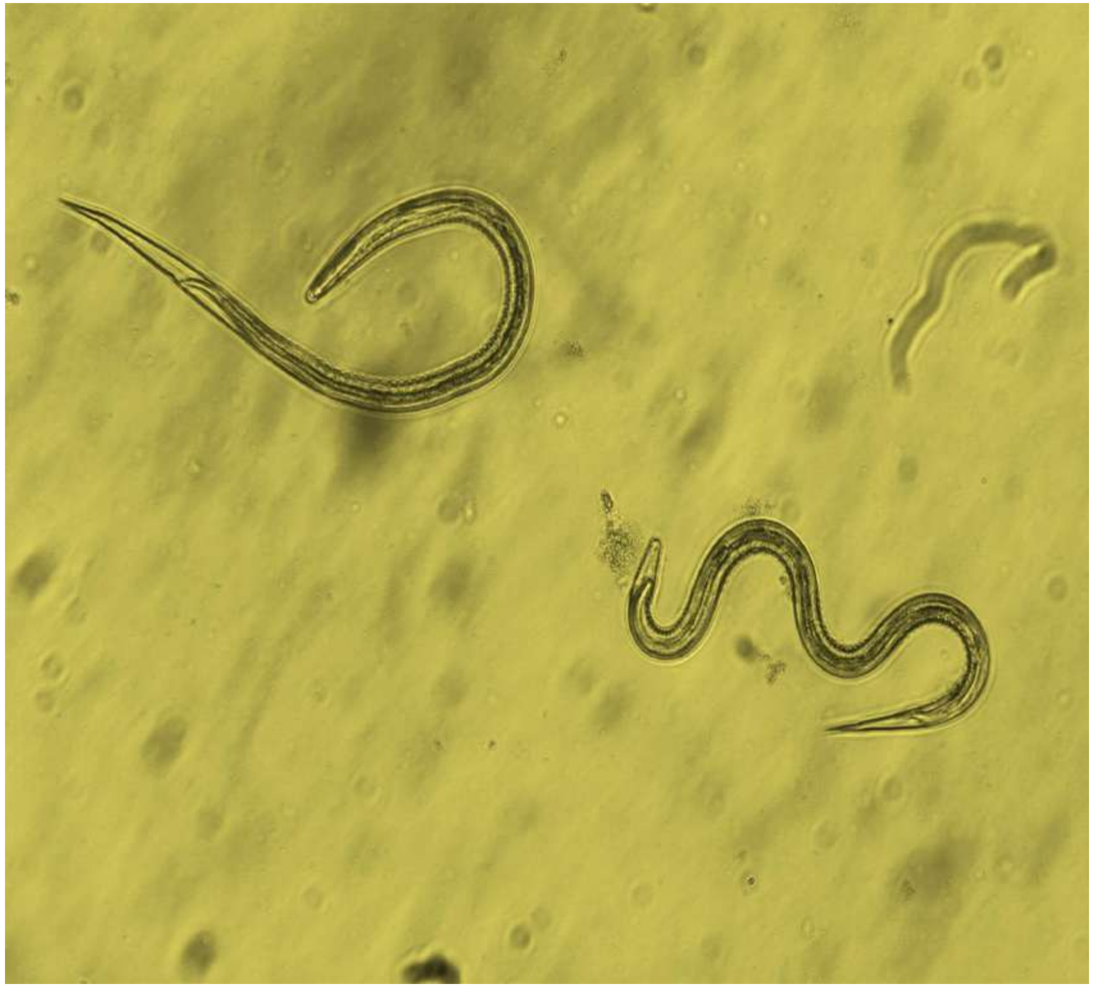

Teladorsagia circumcincta L3s without treatment neither by Eos nor by anthelmintic drugs as negative control (25X magnification).

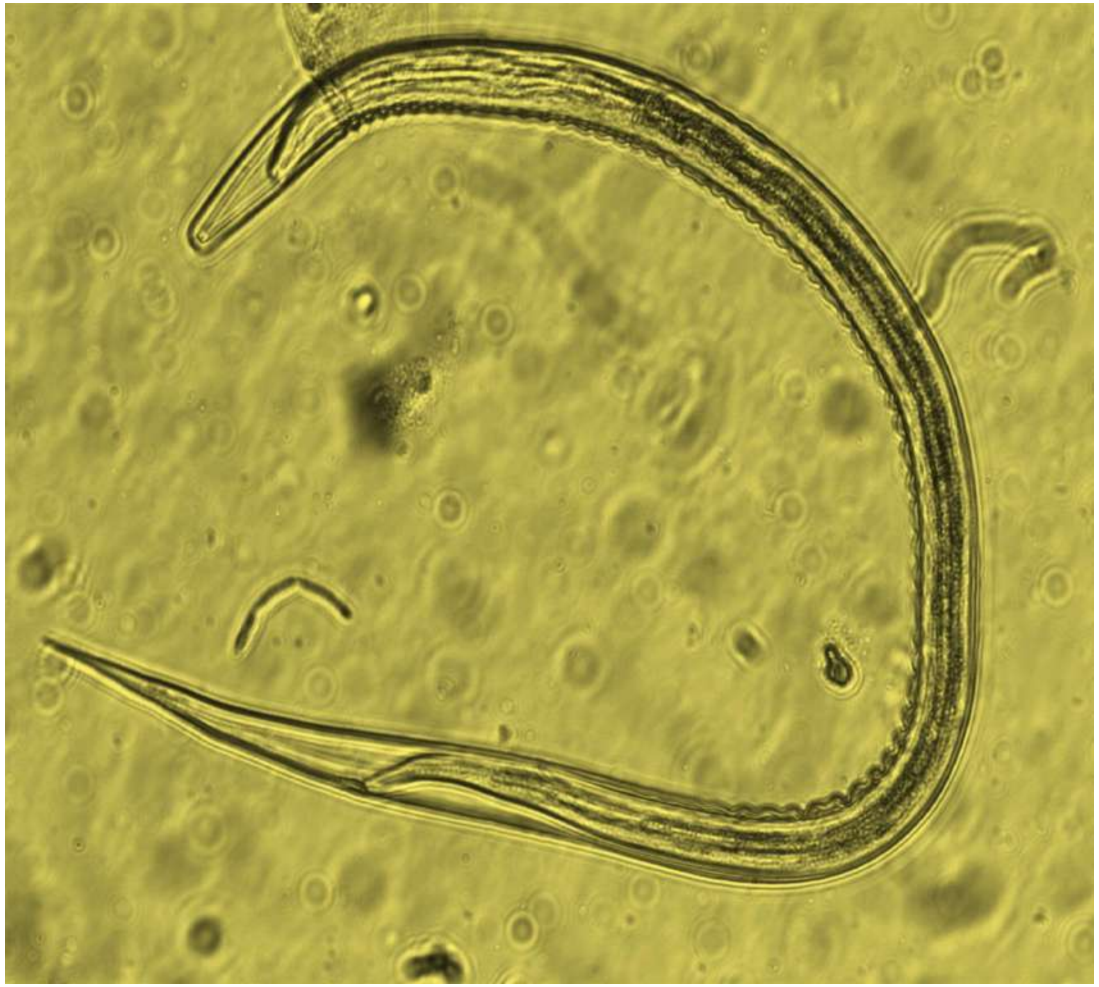

Trichostrongylus vitrinus L3s after treatment with Coriander and linalool 2% (10X magnification). Dead L3s are immobile upon prodding

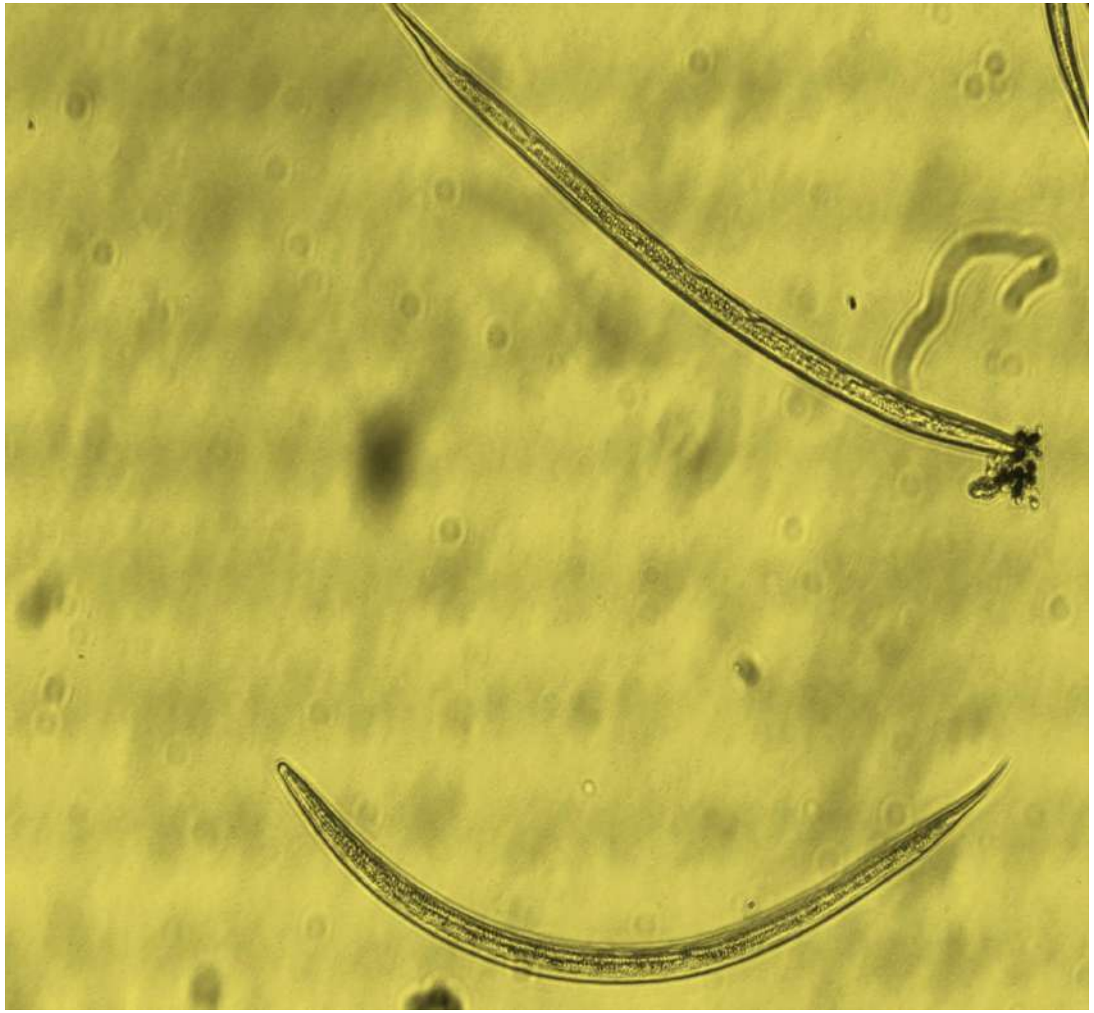

Trichostrongylus vitrinus L3s after treatment with Coriander and linalool 0.125% (10X magnification). Alive L3s are motile upon prodding

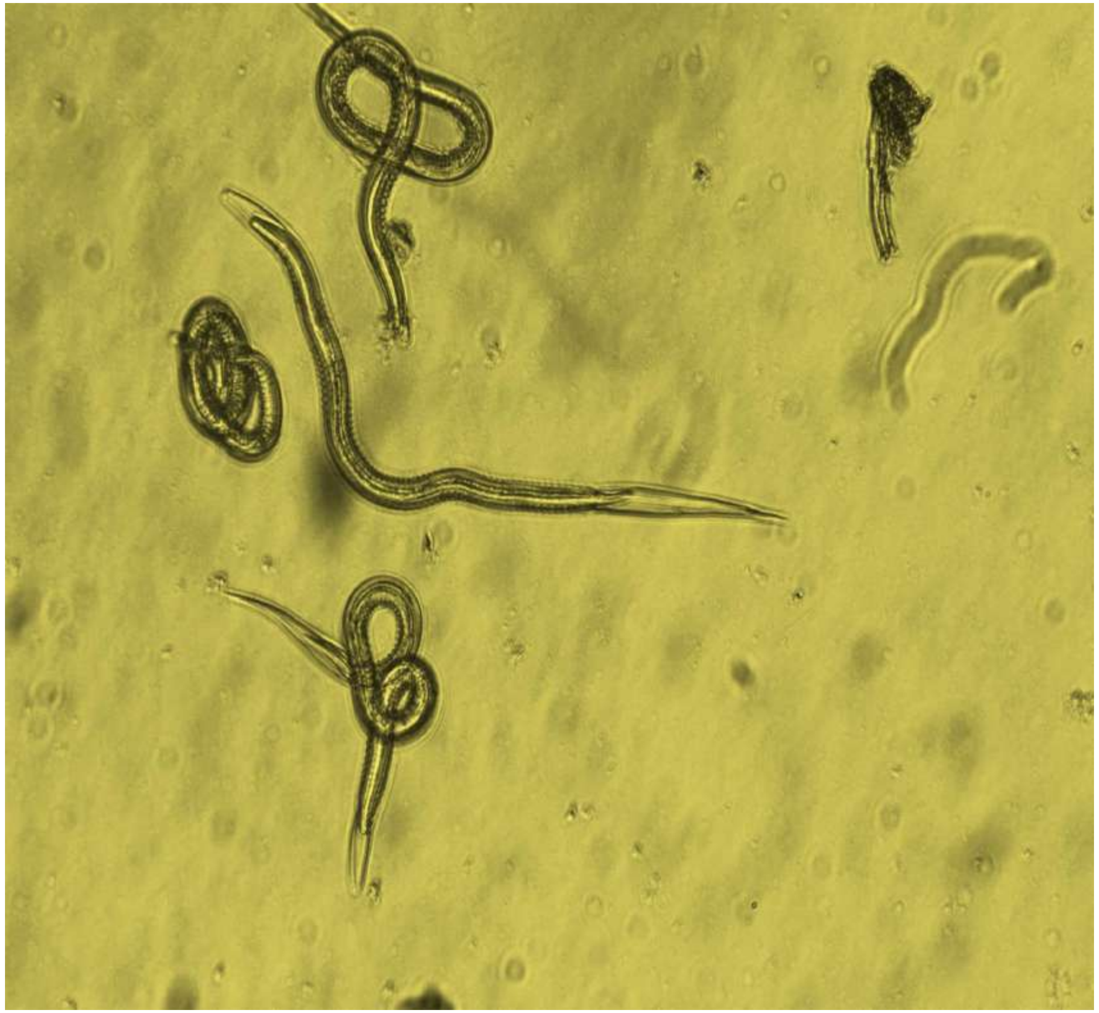

Trichostrongylus vitrines L3s after treatment with levamisole 20 mg/ml as positive control 1 (10X magnification).

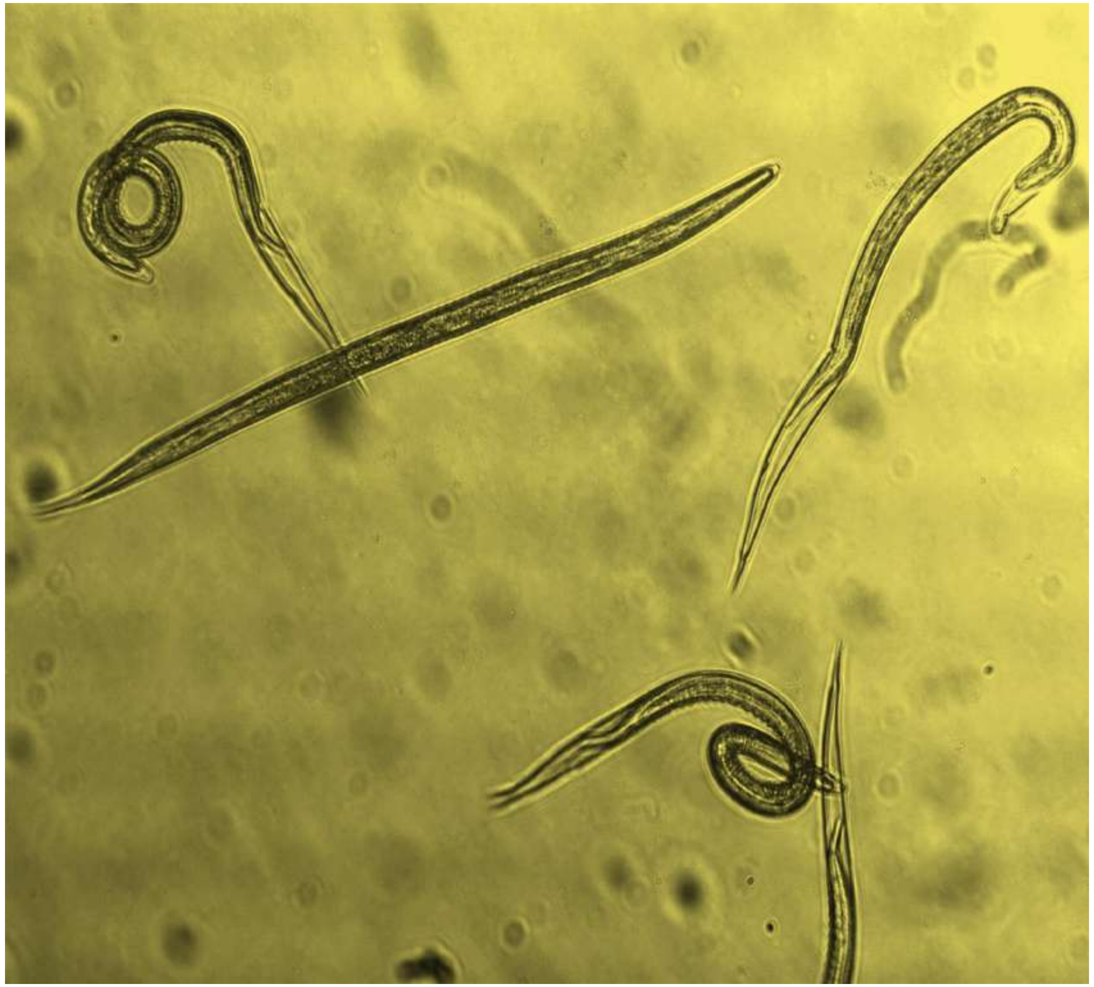

Trichostrongylus vitrinus L3s after heating treatment at 70 C for 10 minutes as positive control 2 (10X magnification). The dead Dead L3s are immobile upon prodding.

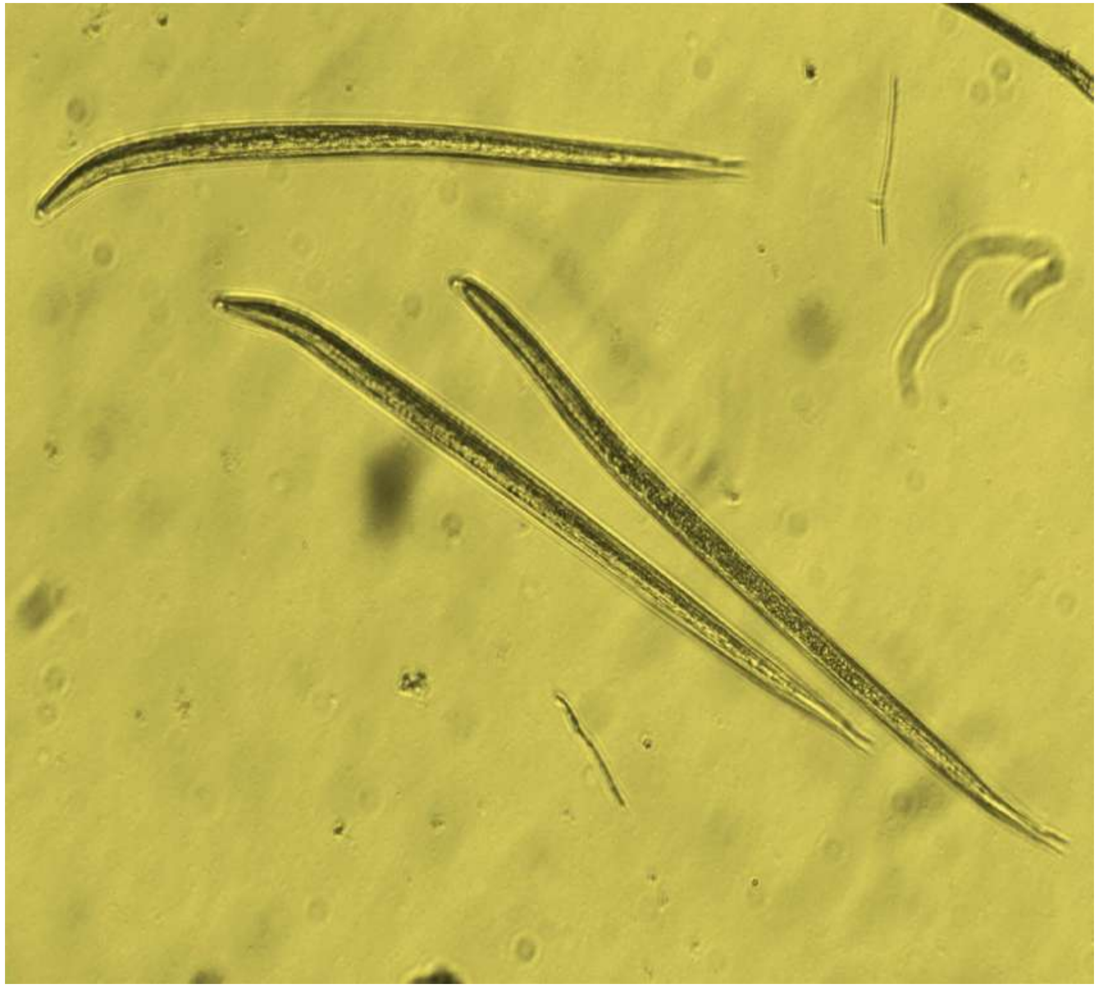

Trichostrongylus vitrines L3s without treatment neither by Eos nor by anthelmintic drugs as negative control (10X magnification).

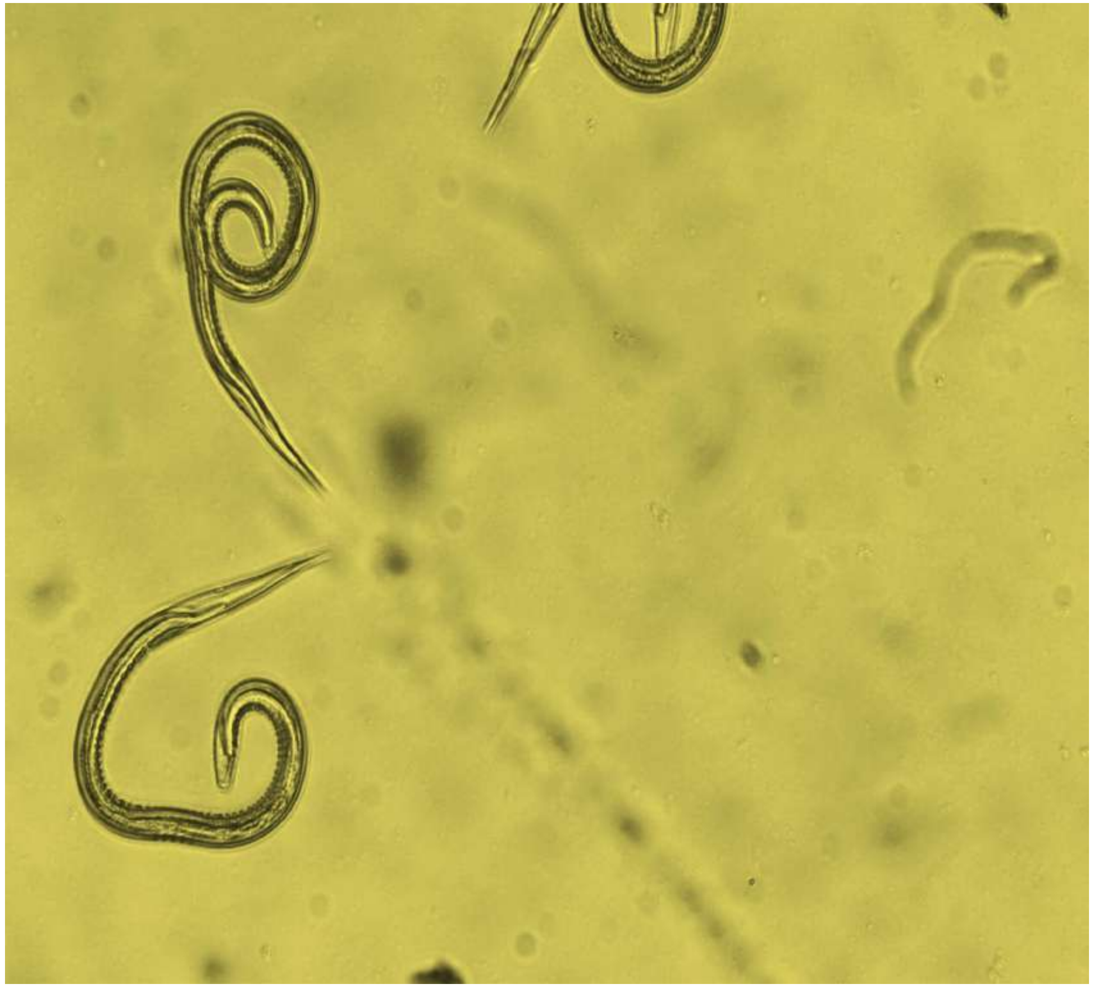

Trichostrongylus vitrines L3s without treatment neither by Eos nor by anthelmintic drugs as negative control (25X magnification).

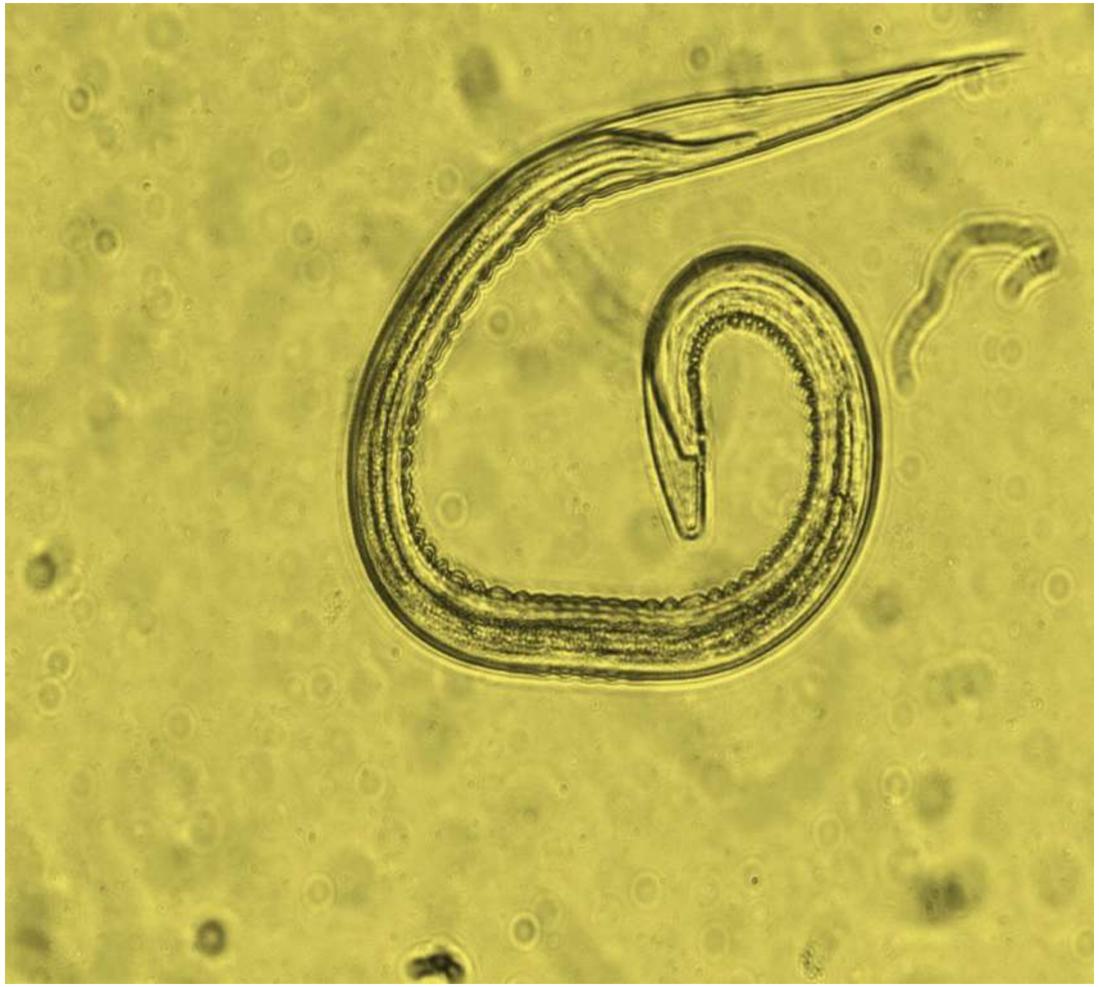

Cooperia oncophora L3s after treatment with Coriander and linalool 2% (10X magnification). Dead L3s are immobile upon prodding

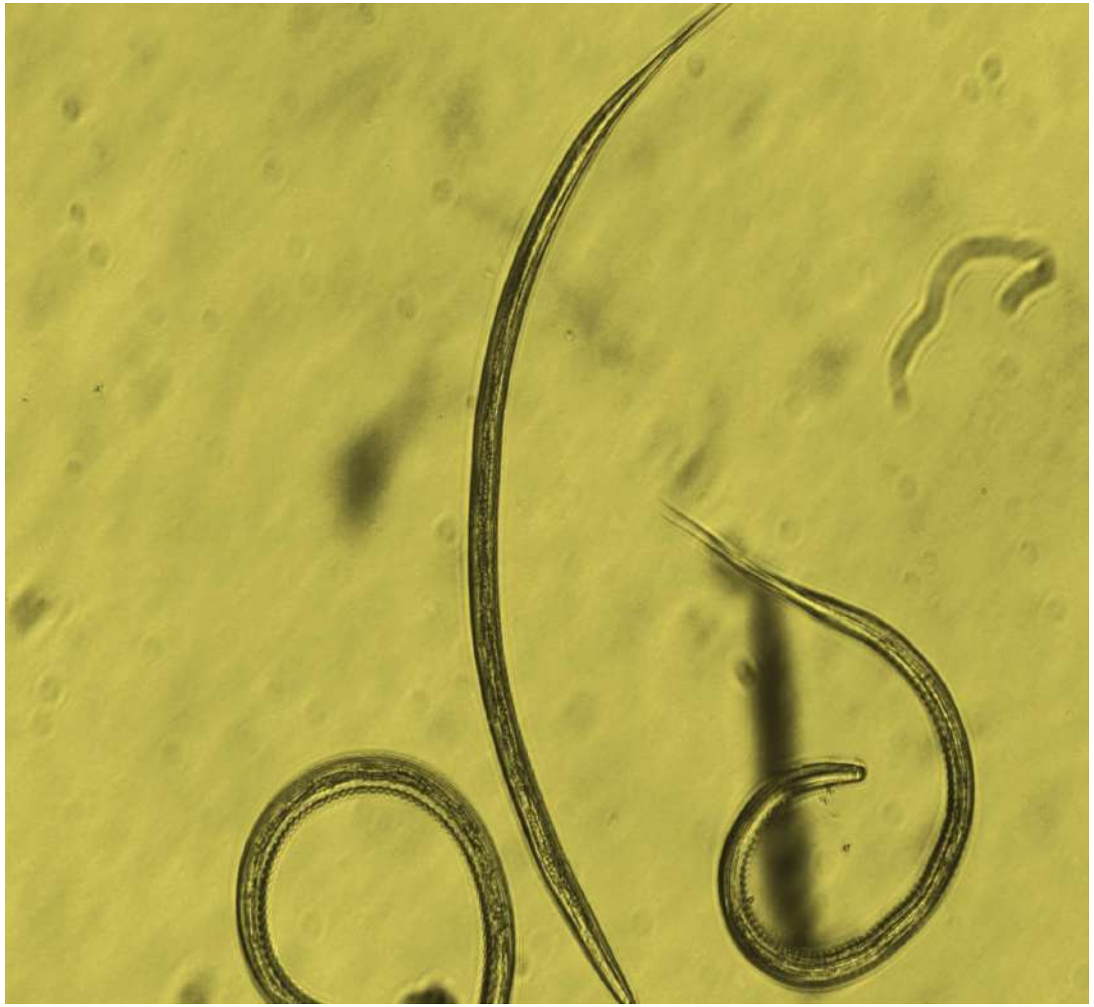

Cooperia oncophora L3s after treatment with Coriander and linalool 0.125% (10X magnification). Alive L3s are motile upon prodding

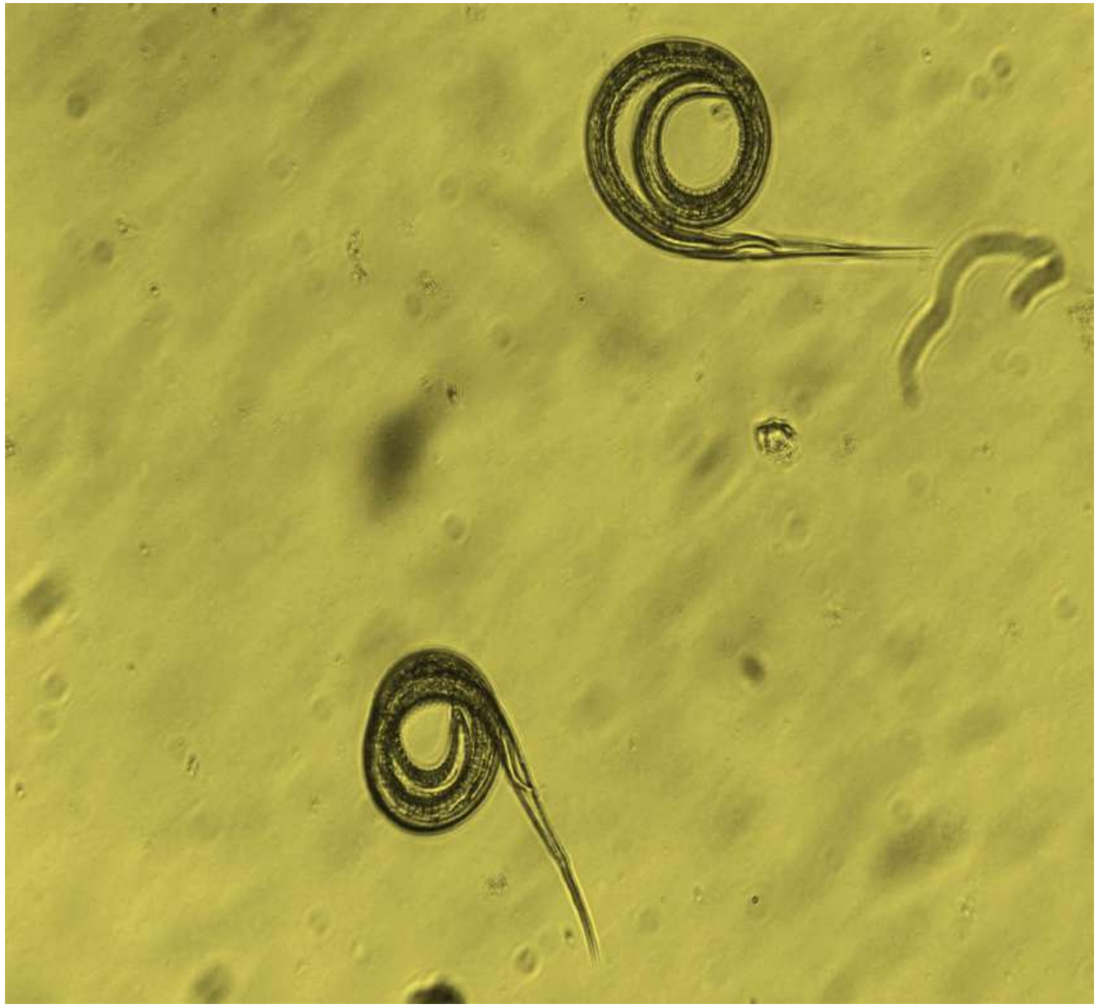

Cooperia oncophora L3s after treatment with levamisole 20 mg/ml as positive control 1 (10X magnification).

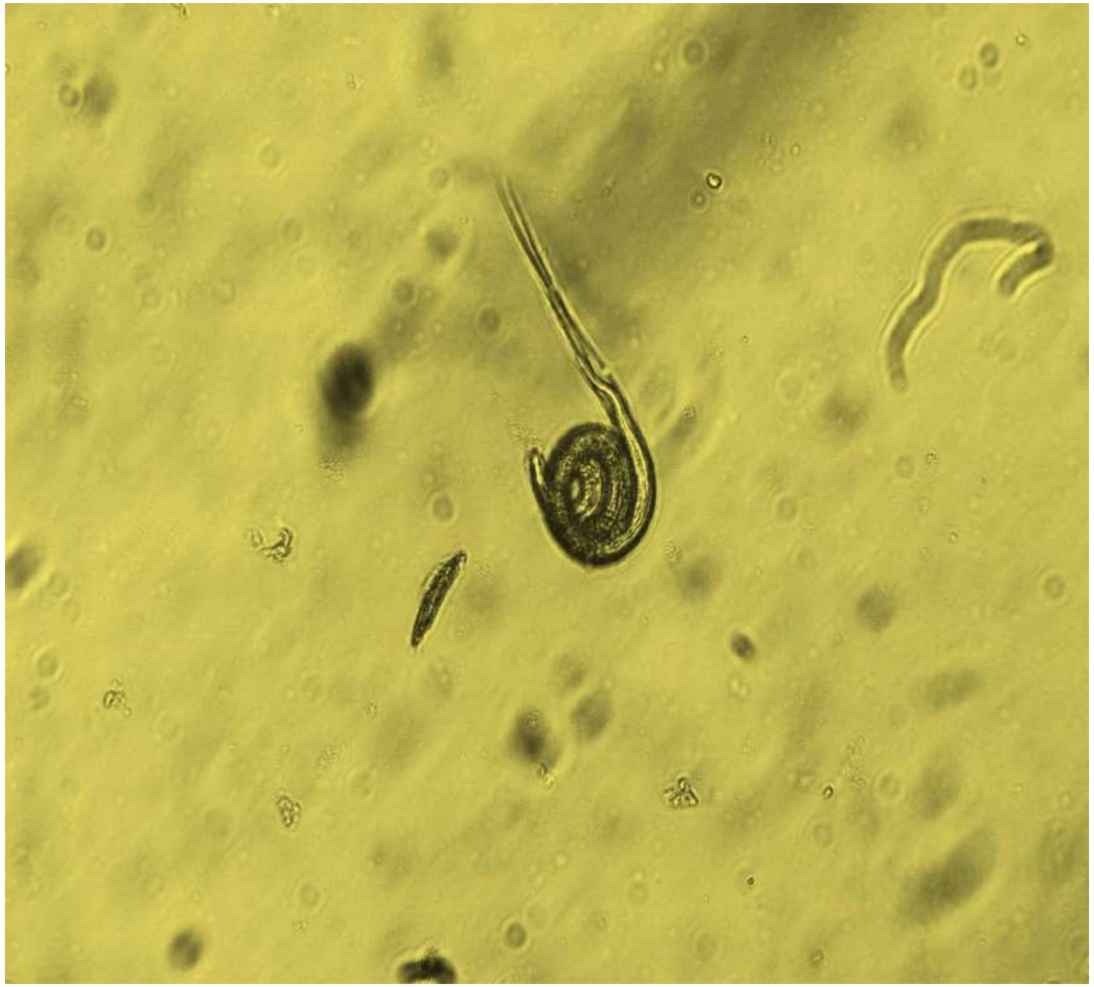

Cooperia oncophora L3s after heating treatment at 70 C for 10 minutes as positive control 2 (10X magnification). The dead Dead L3s are immobile upon prodding.

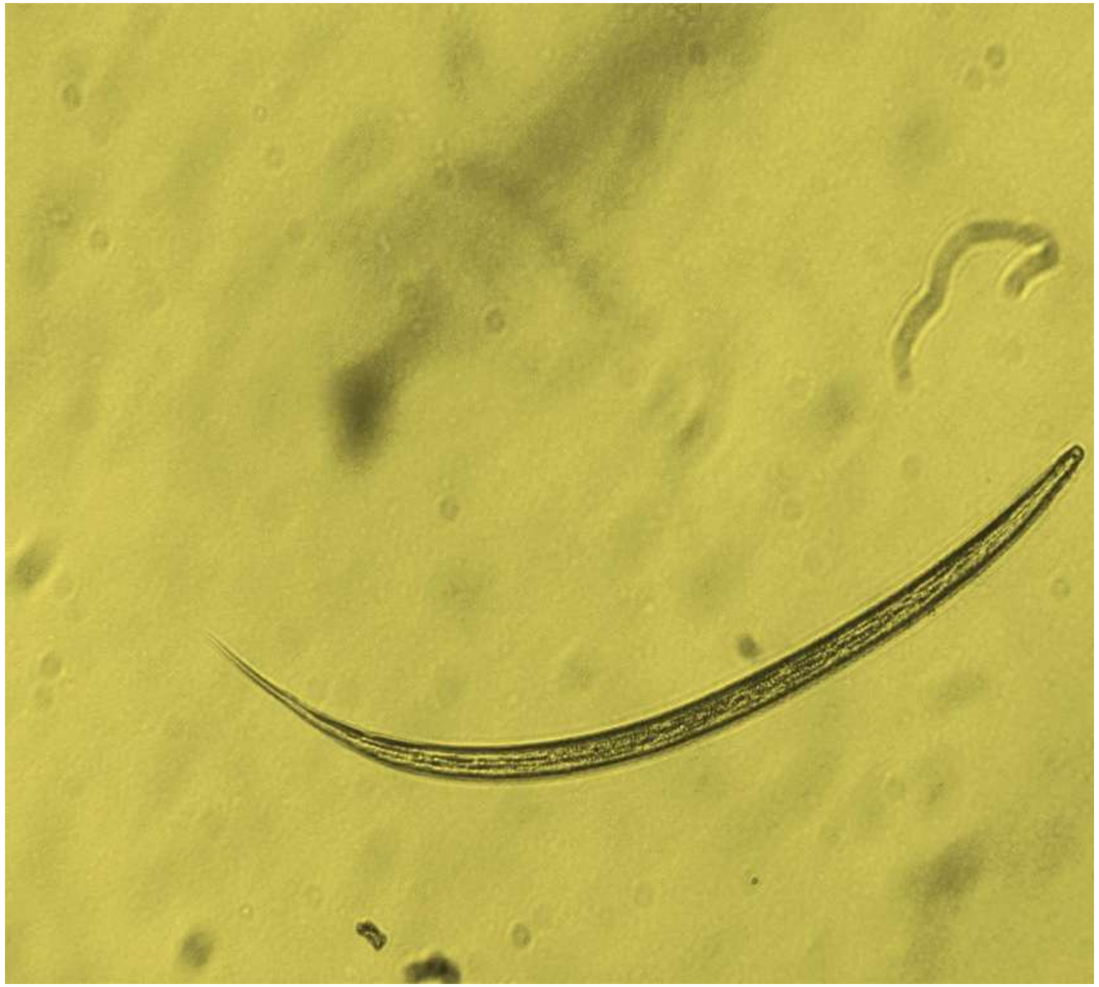

Cooperia oncophora L3s without treatment neither by Eos nor by anthelmintic drugs as negative control (10X magnification).

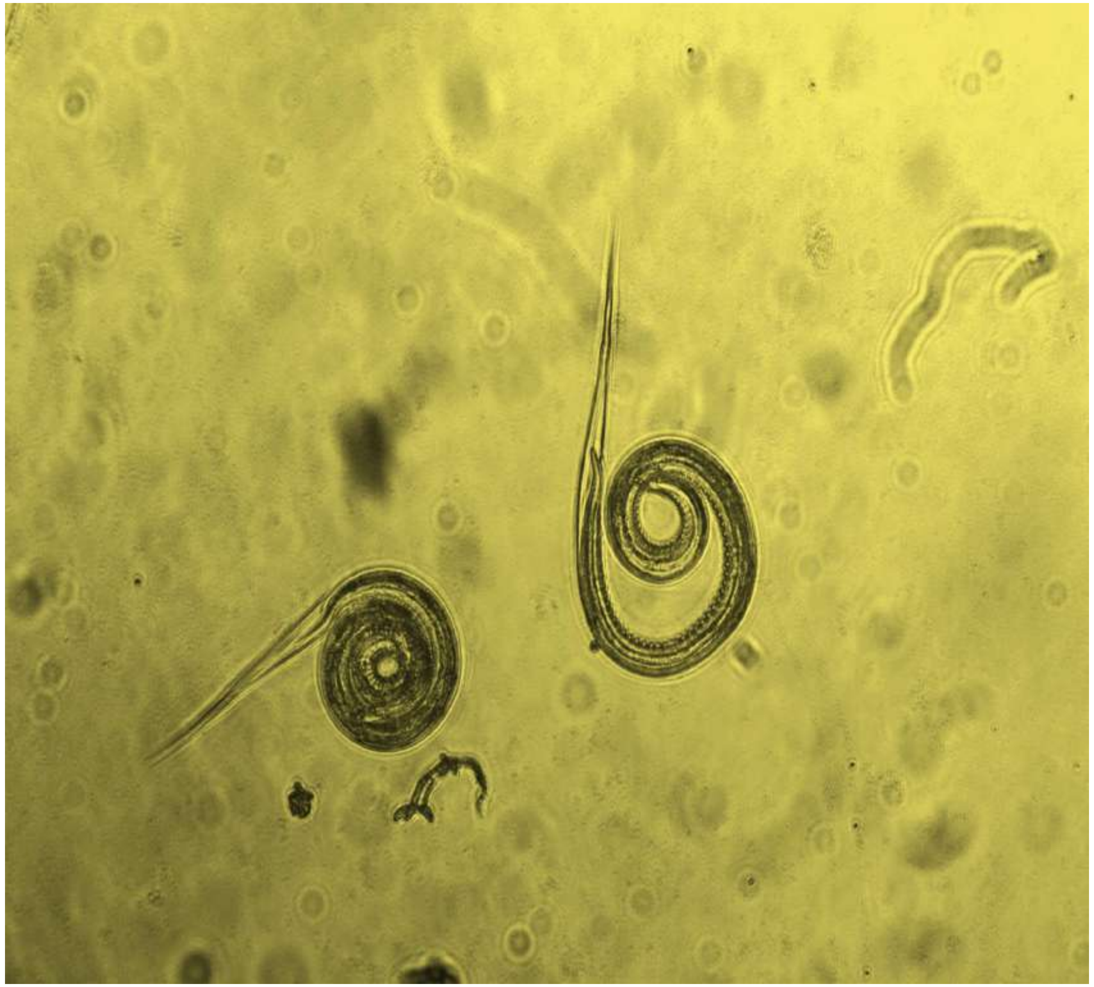

Cooperia oncophora L3s without treatment neither by Eos nor by anthelmintic drugs as negative control (25X magnification).

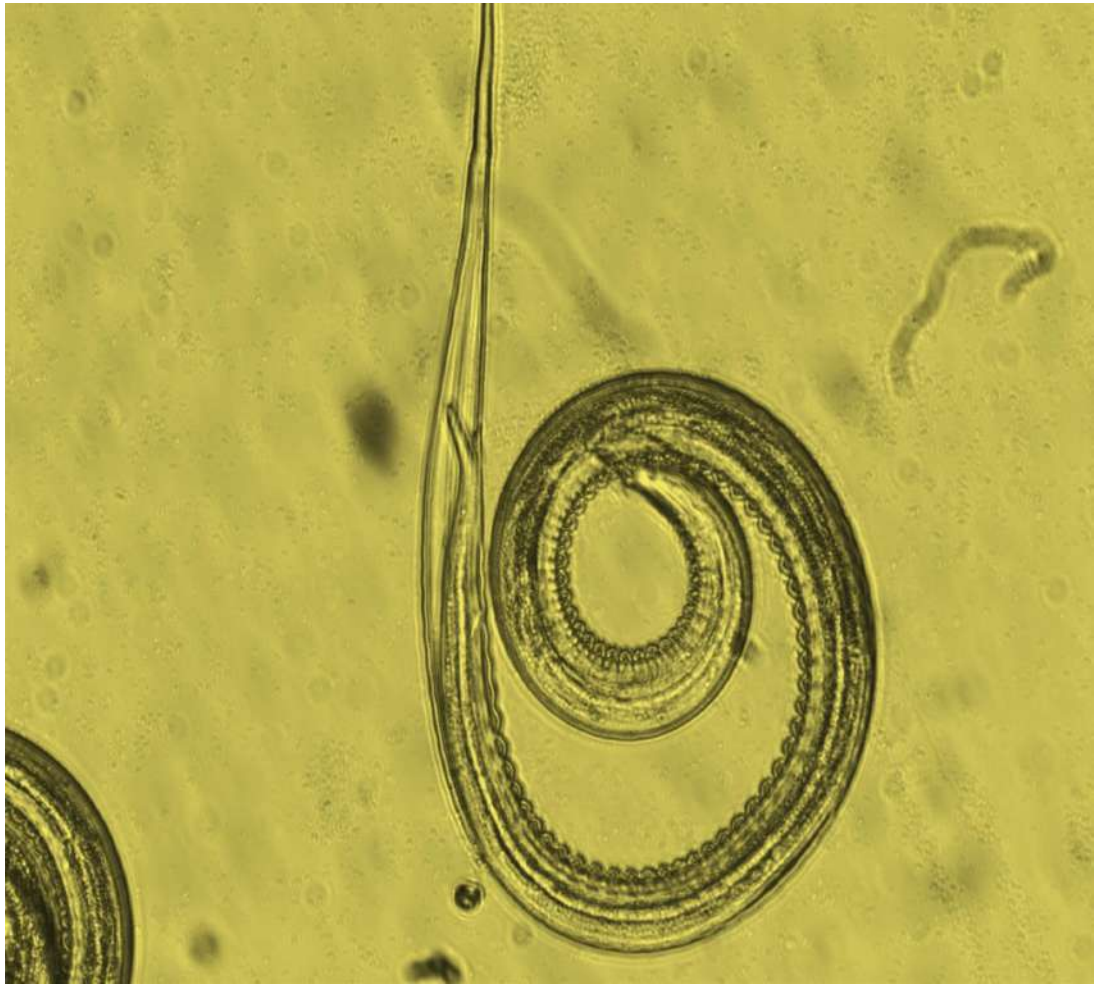

Supplement: Supplementary file 1 [file pathogens-09-00740-s001.zip › Supplementary materials/Figure S1 .pdf]
